# The Tnt1 Retrotransposon Escapes Silencing in Tobacco, Its Natural Host

**DOI:** 10.1371/journal.pone.0033816

**Published:** 2012-03-30

**Authors:** Inmaculada Hernández-Pinzón, Marta Cifuentes, Elizabeth Hénaff, Néstor Santiago, M. Lluïsa Espinás, Josep M. Casacuberta

**Affiliations:** 1 Department of Molecular Genetics, Center for Research in Agricultural Genomics, CRAG (CSIC-IRTA-UAB), Barcelona, Spain; 2 Department of Molecular Genomics, Molecular Biology Institute of Barcelona (IBMB), CSIC, Barcelona, Spain; University of Leeds, United Kingdom

## Abstract

Retrotransposons' high capacity for mutagenesis is a threat that genomes need to control tightly. Transcriptional gene silencing is a general and highly effective control of retrotransposon expression. Yet, some retrotransposons manage to transpose and proliferate in plant genomes, suggesting that, as shown for plant viruses, retrotransposons can escape silencing. However no evidence of retrotransposon silencing escape has been reported. Here we analyze the silencing control of the tobacco Tnt1 retrotransposon and report that even though constructs driven by the Tnt1 promoter become silenced when stably integrated in tobacco, the endogenous Tnt1 elements remain active. Silencing of Tnt1-containing transgenes correlates with high DNA methylation and the inability to incorporate H2A.Z into their promoters, whereas the endogenous Tnt1 elements remain partially methylated at asymmetrical positions and incorporate H2A.Z upon induction. Our results show that the promoter of Tnt1 is a target of silencing in tobacco, but also that endogenous Tnt1 elements can escape this control and be expressed in their natural host.

## Introduction

Mobile genetic elements are present in virtually all genomes where they occupy a variable but very high proportion. In plants they can account from 21% of the genome as in *Arabidopsis thaliana* [1] to as much as 85% in the case of maize [2]. While transposons are a very rich source of new genic functions and regulatory mechanisms, and thus a motor of evolution [3], transposition remains a potentially highly mutagenic event that genomes need to keep under tight control. This is particularly true for retrotransposons which, due to their replicative mechanism of transposition, have the capacity to invade genomes and reach very high copy numbers. Genomes have therefore developed sophisticated methods to control transposition, the most general and efficient of which is likely to be gene silencing. In fact, it has long been proposed that homology dependent gene silencing mechanisms and, in general, the epigenetic mechanisms that control gene expression in complex genomes, have evolved from host defense mechanisms against parasitic sequences such as viruses and transposons [4]. While viruses are controlled by post-transcriptional gene silencing (PTGS) [5], transposons are the primary target of transcriptional gene silencing (TGS) [6], [7]. However, in spite of the high efficiency of these silencing mechanisms, viruses and transposons have continued to proliferate, probably because they have evolved mechanisms to escape host control. Indeed, in the last few years a number of viral proteins, known as silencing suppressors, with the capacity to block silencing at different levels have been described [8], [9]. Viruses have thus developed active mechanisms to counteract host defenses and escape tfrom host control. Strikingly, no active mechanisms that could parallel the viral strategies to avoid silencing have been described yet for transposons.

Most retrotransposons are highly silenced in plants and are only reactivated in certain mutant backgrounds or under severe stress conditions. Cell culture stresses can reactivate epigenetically-silenced retrotransposons such as *LORE1* from *Lotus japonicus* [10] or *Tos17* [11] and other rice elements [12], and it has been recently shown that interfering with the epigenetic machinery can also reactivate and induce proliferation of silent retroelements [13], [14]. Genomes manage to differentiate between transposable elements and endogenous genes and silence specifically the former, probably because transposons are more prone to produce aberrant RNAs [15]. Their multicopy nature increases the chances of read-through transcription generating antisense transcripts, which can also arise from nested insertions which are very frequent in the case of retrotransposons. The possibility that a transposon produces aberrant RNAs increases drastically with its copy number, which makes the more active and high copy number transposons the more highly repressed epigenetically [15]. Under this scenario, retrotransposition is blocked when copy number reaches the threshold of detection and triggers silencing of the whole family. This would explain why low copy number elements tend to be highly expressed when compared to those present at high copy number [16], [17]. Interestingly, when the tobacco retrotransposons Tto1 and Tnt1 are introduced in *Arabidopsis* they can transpose, but become silenced and heavily methylated when their copy number increases [18], [19]. In the case of Tnt1 expression can be reactivated when its copy number is decreased to two by segregation [19] which emphasizes how tightly retrotransposon copy number is controlled by transcriptional gene silencing and how sensitive this mechanism is to copy number.

While epigenetic silencing of transposons is generally very efficient, a handful of elements remain active even when present in high copy numbers in plant genomes. One of the best studied plant retrotransposons, the tobacco Tnt1 element, is present in hundreds of copies and still an important fraction of them remain transcriptionally active [20], [21]. Tnt1 is expressed during pathogen-related stresses due to the presence of a defense-inducible promoter in its long terminal repeat (LTR) [22], [23]. The simultaneous expression of a high number of Tnt1 elements in defense-related stresses suggests that Tnt1, in spite of being present in a high number of copies in tobacco, is not silenced in these particular situations. Here we analyze two alternative hypotheses that could explain this expression. First, TGS could be partially relieved during this particular stress allowing Tnt1 elements to be expressed, or second, the Tnt1 element in particular could be resistant to silencing. The results presented here suggest that TGS is maintained in the stress situations in which Tnt1 is transcribed and show that, although the Tnt1 promoter is a target of TGS mechanisms, endogenous Tnt1 elements manage to escape silencing and be expressed.

## Results

### 1. Effect of defense-related stress on transcriptional silencing

**Figure 1:**
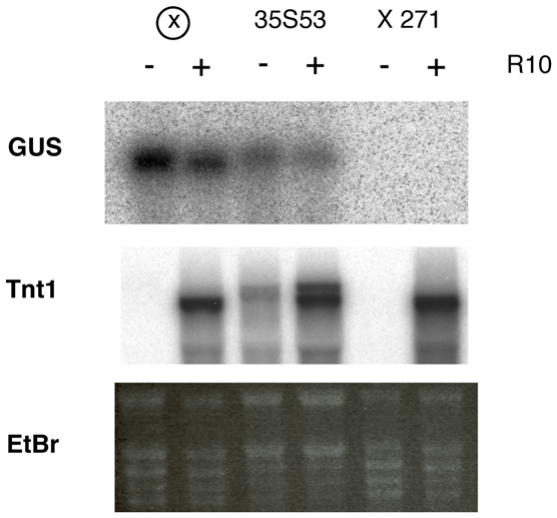
TGS is not alleviated in stress situations that induce Tnt1. A 35S promoter driving a GUS reporter gene stably introduced in tobacco plants was silenced by crossing with a plant containing the 271 silencer locus (×271) or by agro-infiltration with a construct expressing a hairpin of the 35S promoter sequence (35S35). The figure presents northern analysis of RNA obtained from leaves of control and silenced plants either non-treated (−) or treated (+) with the fungal cellulose Onozuka R10 (R10) to induce Tnt1 expression, and hybridized with a GUS probe (upper panels) or a Tnt1 probe (lower panels). Agroinfiltration with the hairpin 35S construct produces a basal induction of Tnt1 , due to a defense-related reaction against *Agrobacterium tumefaciens*. An image of the EtBr staining of the RNA gel used is shown underneath as loading control.

Plant retrotransposons, and mobile genetic elements in general, are usually silent and those which can be expressed are only active in particular situations. Plant retrotransposon expression has been shown to be induced by cell culture [10], [12], [24], defense-related stresses [22], [25], and heat stress [26] and there is a longstanding view that plant retrotransposon activation is associated with stress [27], [28]. This association may suggest a release of the general control mechanisms, and in particular TGS, in stress situations. Indeed, the reactivation of silent retrotransposons in cell culture is often associated with a decrease in methylation of these elements [10], [11] suggesting a decrease of their TGS control in cell culture. Similarly, it has been recently shown that heat stress can activate heterochromatic transcription [29], and also that several types of stresses can affect DNA methylation in plants [30], [31]. The expression of the tobacco retrotransposon Tnt1 produces 2 different transcripts of 5.2 and 6.5 knt. However only the 5.2 knt transcript is considered as the retrotransposon transcript, the one of 6.5 knt probably being the result of the expression of a particular Tnt1 copy from an external unrelated promoter [32]. The expression of the tobacco Tnt1 retrotransposon is induced by defense-related stress [22]. We have therefore investigated whether TGS is alleviated globally in those defense-related stress situations. In all subsequent experiments, this stress is mimicked by leaf infiltration with the fungal cellulose extract Onozuka R10 (hereafter referred as R10), which induces Tnt1 expression in the whole leaf tissue [33], [34]. First we have analyzed the transcriptional silencing of the 35S promoter in a silencing background under normal and stress situations. We used the 271 locus which contains a complex array of a nitrate reductase coding sequence placed under the control of a 35S promoter and, as a consequence, silences the nitrate reductase gene post-transcriptionally, as well as any 35S promoter sequence longer than 90 nt by TGS [35]. Transgenic plants containing a 35S-GUS construct express the GUS reporter gene in a constitutive manner and its expression is not changed by treatment with R10, which induces the expression of the Tnt1 retrotransposon (Figure 1). As expected, when these plants were crossed with plants containing the 271 *locus*, the expression of the transgene was completely silenced. Upon treatment with R10, which induced the endogenous Tnt1 expression, the expression of GUS was not recovered (Figure 1), showing that silencing was maintained under Tnt1 inducing conditions.

The 271 locus is a strong silencer and it could be argued that a partial release of TGS could be difficult to detect with this experimental design. We thus designed another 35S silencing system which would allow us to detect a limited release of TGS. We used a hairpin-based approach to produce small interfering RNAs (siRNAs) corresponding to the 35S promoter in order to transiently silence a 35S-GUS construct stably introduced in tobacco. Agroinfiltration of a hairpin of the 35S promoter sequences driven by the promoter of the AtBH2 gene, highly active in leaf tissue [36] into the leaves of a 35S-GUS containing plant resulted in the transient and partial transcriptional silencing of the 35S promoter. The silencing of the transgene, shown by a decrease of GUS mRNA accumulation, was detectable one day after infiltration and progressed until 5 days (Figure S1A). The silencing was accompanied by increased methylation of the promoter sequences (Figure S1B). Comparative expression and methylation analyses showed that while both silencing strategies induce a similar level of methylation, the degree of transcriptional gene silencing produced by the 35S hairpin is weaker than the one produced by the 271 locus (Figure S1C and S1D). The analysis of 35S hairpin-silenced plants treated with R10, which induced the transcription of the endogenous Tnt1 elements, showed no recovery of GUS expression (Figure 1).

In summary we have not detected any recovery of the silenced 35S promoter in R10-treated leaves that would indicate a global decrease of TGS and explain Tnt1 expression.

### 2. The Tnt1 promoter is a target of TGS

As a general release of silencing during stress cannot be invoked to explain the expression of the high copy number Tnt1 retrotransposon, we next tested whether the Tnt1 promoter is a target of TGS in tobacco. It has been previously shown that siRNAs corresponding to the Tnt1 LTR are present in tobacco [37]. An analysis of the available tobacco siRNA databases (http://smallrna.udel.edu/) confirmed that tobacco produces siRNAs matching the Tnt1 sequence. The size distribution of the siRNAs complementary to the Tnt1 sequence shows two different peaks at 21–22 and 24 nt (Figure S2A). As 21 and 24 nt siRNAs have been related to PTGS and TGS [38], this suggests that Tnt1 may be a target of both types of silencing. Although there are 24 nt siRNAs directed against sequences within different Tnt1 regions, the vast majority target the LTR region, where the promoter of Tnt1 is located (Figure S2B). This shows that the LTR of Tnt1 is a specific target of 24 nt siRNAs and suggests the existence of an active TGS mechanism directed towards the Tnt1 promoter in tobacco.

**Figure 2:**
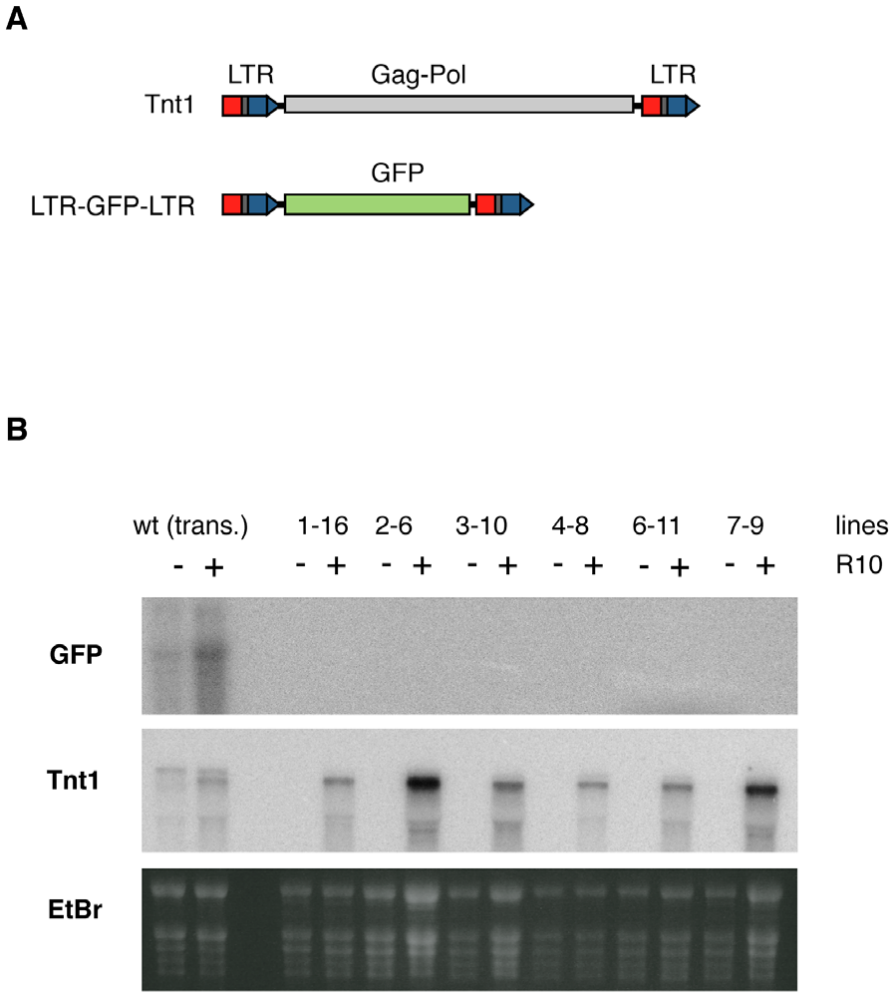
Constructs containing the LTRs of Tnt1 flanking a GFP reporter gene are silenced when stably introduced in tobacco. (A) Scheme of the LTR-GFP-LTR construct and of a typical Tnt1 element for comparison. (B) Northern analyses of GFP and Tnt1 expression in wild type plants agroinfiltrated with the LTR-GFP-LTR construct and expressing it transient expression (trans.), as well as in plants stably transformed with the same construct (the name of the transgenic line is indicated on the top). RNA was extracted from leaves non-treated (−) and treated (+) with R10 and subsequently hybridized with a GFP probe (upper panel) or a Tnt1 probe (middle panel). An image of the EtBr staining of the RNA gel used is shown as loading control (lower panel).

To determine whether the Tnt1 promoter can be efficiently silenced in tobacco we analyzed the expression of a construct (LTR-GFP-LTR) in which a GFP reporter gene was flanked by two Tnt1 LTRs, providing the promoter and terminator sequences, in an arrangement that resembles that of the endogenous Tnt1 elements (Figure 2A). While this construct was expressed as expected in transient expression assays, it was completely silenced when stably introduced into tobacco (Figure 2B). Indeed, none of the 15 independent transgenic lines analyzed expressed the transgene in the conditions where the endogenous Tnt1 elements are induced (Figure 2B shows the analysis of 6 representative lines). The infiltration of the transgenic plants with a construct expressing the HcPro viral suppressor of PTGS [39] prior to the induction of Tnt1 did not affect the expression of the transgene (Figure S3), suggesting that the transcription of the transgene was silenced rather by TGS.

The analysis of other constructs containing fragments of the Tnt1 LTR together with fragments of the 35S promoter gave a similar result. While these constructs were expressed as expected in transient expression assays (not shown), they were silenced when stably introduced in tobacco (Figure S4). As expected, the degree of silencing of these transgenic lines containing small fragments of the Tnt1 LTR was lower compared to the silencing of the LTR-GFP-LTR transgene which contains two complete LTRs. The degree of silencing varied among the different promoters and in some cases it also varied among different individuals, showing a high degree of stochasticity (Figure S4). This result confirms that the sequences of the LTR of Tnt1 are a target of TGS in tobacco.

**Figure 3:**
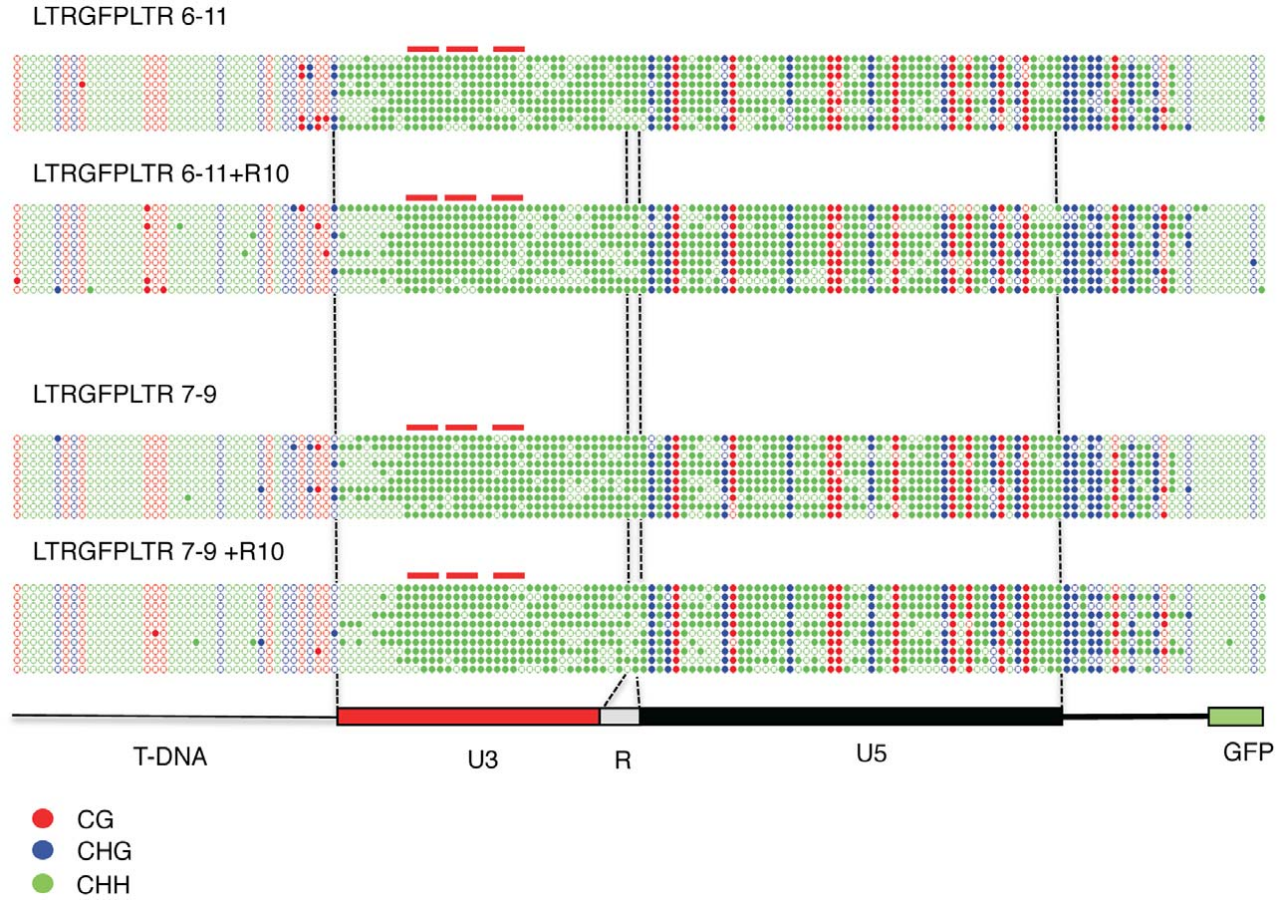
LTR-GFP-LTR transgenes are highly methylated in both symmetrical and asymmetrical contexts. The 5′ region of the LTR-GFP-LTR transgene, including the 5′ LTR, was amplified and cloned from bisulfite converted DNA from R10-treated (+ R10) as well as non-treated leaves of two independent transgenic lines. At least 10 clones were sequenced from each transgenic line (only one sequence is shown when the same sequence was obtained several times). The methylation state of each cytosine is shown as open (not methylated) or closed (methylated) circle. The sequence context of each cytosine is shown by the color of the circle (red, CG; blue, CHG; green, CHH). The different regions of the transgene are shown. The position of the BII boxes, known to bind defense-induced DNA binding factors [59] are indicated by red lines.

In plants TGS is usually accompanied by DNA methylation of the silenced promoters, in particular in the case of transposable elements [40]. We have therefore analyzed the level of methylation of the promoter sequences within the silenced LTR-GFP-LTR plants by bisulfite sequencing. Our results showed that although the T-DNA and the GFP sequences present in the transgene are essentially free of methylation, the transgene sequences showing sequence identity to the endogenous Tnt1 elements (i.e. the LTR sequences) are heavily methylated in both symmetrical and asymmetrical methylation contexts (Figure 3). The degree of methylation is similar in leaves treated and non-treated with R10. The degree of methylation is also similar in the 5′ and the 3′ LTRs (compare Figure 3 with Figure S5). Our analysis shows that more than 80% of the cytosines located in a symmetrical context are methylated while methylation at asymmetrical positions ranges from 70 to 77%.

The results presented here clearly show that Tnt1 promoter sequences are a target of TGS in tobacco and that they can be highly methylated and efficiently silenced when present in reporter constructs stably introduced in tobacco.

### 3. Analysis of the expression and methylation of endogenous Tnt1 elements

**Figure 4:**
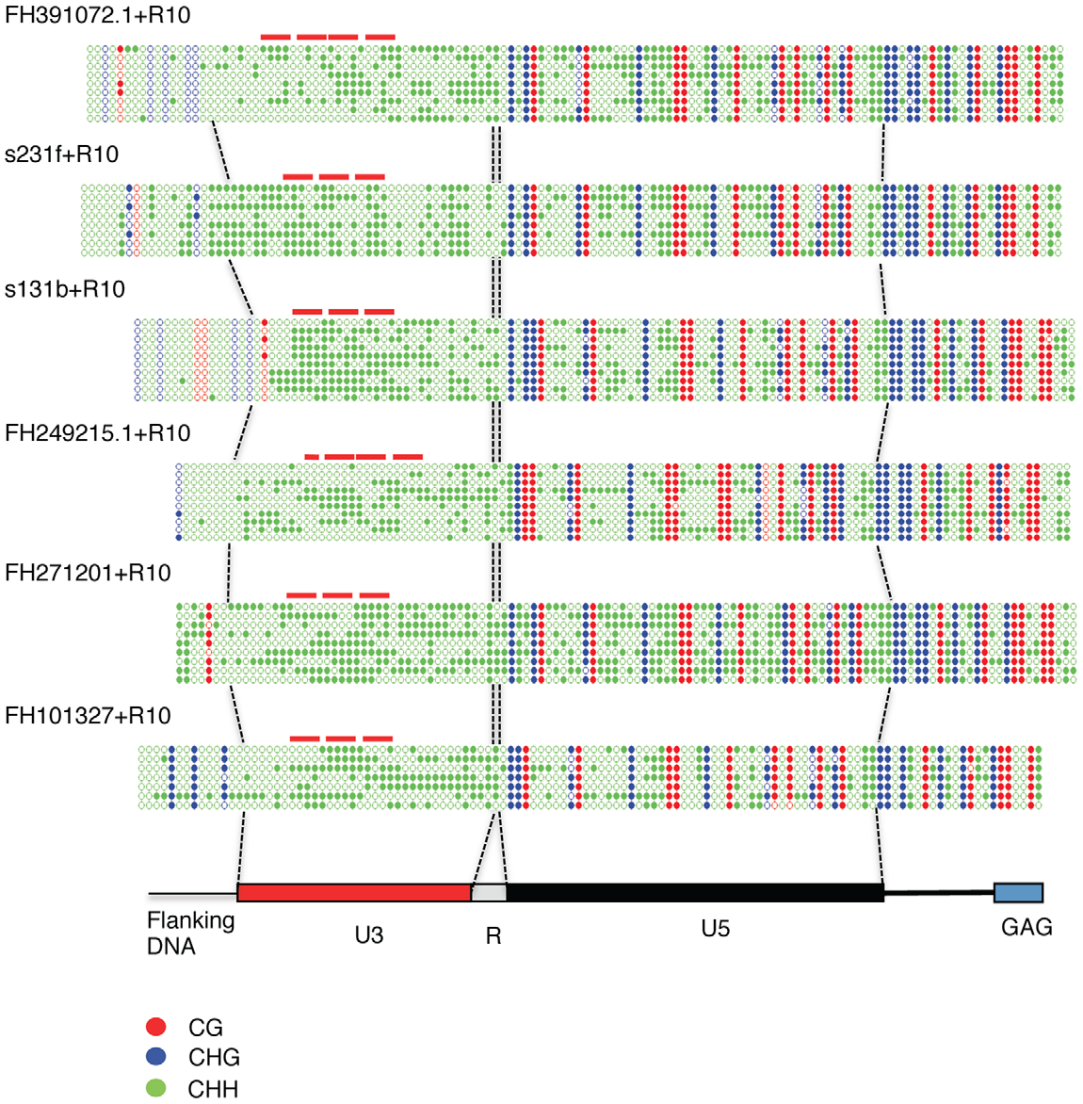
DNA methylation status of endogenous Tnt1 elements. The 5′ region of 6 endogenous Tnt1 elements, including the 5′ LTR, was amplified and cloned from bisulfite converted DNA of R10-treated leaves. At least 10 clones were sequenced from each endogenous Tnt1 element (only one sequence is shown when the same sequence was obtained several times). The methylation state of each cytosine is shown as in Figure 3. The different regions of the Tnt1 elements are shown. The position of the BII boxes, known to bind defense-induced DNA binding factors [59] are indicated by red lines.

Tnt1 is present in hundreds of copies in tobacco, an important fraction of which is transcriptionally competent [20], [21]. However, there are at least three different Tnt1 subfamilies that are differentially regulated, and only a subset of the Tnt1 elements is expected to be expressed under particular stress conditions [23]. Treatments with R10 induce mainly the Tnt1A subfamily [22], [41]. Therefore, we decided to analyze the methylation state of the promoters of Tnt1A elements induced by R10. In order to select potentially active and R10 inducible Tnt1 elements we compared the sequences of 157 Tnt1 LTRs present in public databases (mainly as Genome Survey Sequences, GSS) with 25 Tnt1 mRNA sequences obtained from R10 induced leaves [41] A phylogenetic analysis of these sequences showed that a subset of the genomic LTR sequences clusters together with those from experimentally obtained RNA from Tnt1 expressed elements (Figure S6). We selected 6 different endogenous Tnt1 elements among those showing the highest sequence similarity to R10-induced Tnt1 RNA sequences (shown by a red arrow in Figure S6) and tested the methylation profile of their LTRs by bisulfite sequencing. Methylation of the few symmetrical positions present in the analyzed region was found to be very high (between 93 and 100% at CG positions and between 75 and 95% at CHG positions). On the contrary, methylation levels at asymmetrical positions were relatively low, ranging from 32 to 51% (Figure 4). The degree of methylation was similar in plants not treated with R10 (not shown). An analysis of the methylation at the 3′LTR of one of the endogenous elements showed that both LTRs seem to have the same methylation patterns, with high methylation at symmetrical positions and limited methylation at asymmetrical ones (Figure S7). A Southern analysis of tobacco DNA digested with methylation-sensitive enzymes shows that the whole population of endogenous Tnt1 elements can be efficiently digested with enzymes blocked by asymmetrical methylation, while being completely resistant to digestion with enzymes blocked by symmetrical methylation (Figure S8). This result indicates that endogenous Tnt1 elements are heavily methylated at symmetrical positions, while methylation is much less frequent at asymmetrical ones, and suggests that the pattern of methylation found for the 6 selected potentially active Tnt1 elements is a general trend of the tobacco Tnt1 population.

**Figure 5:**
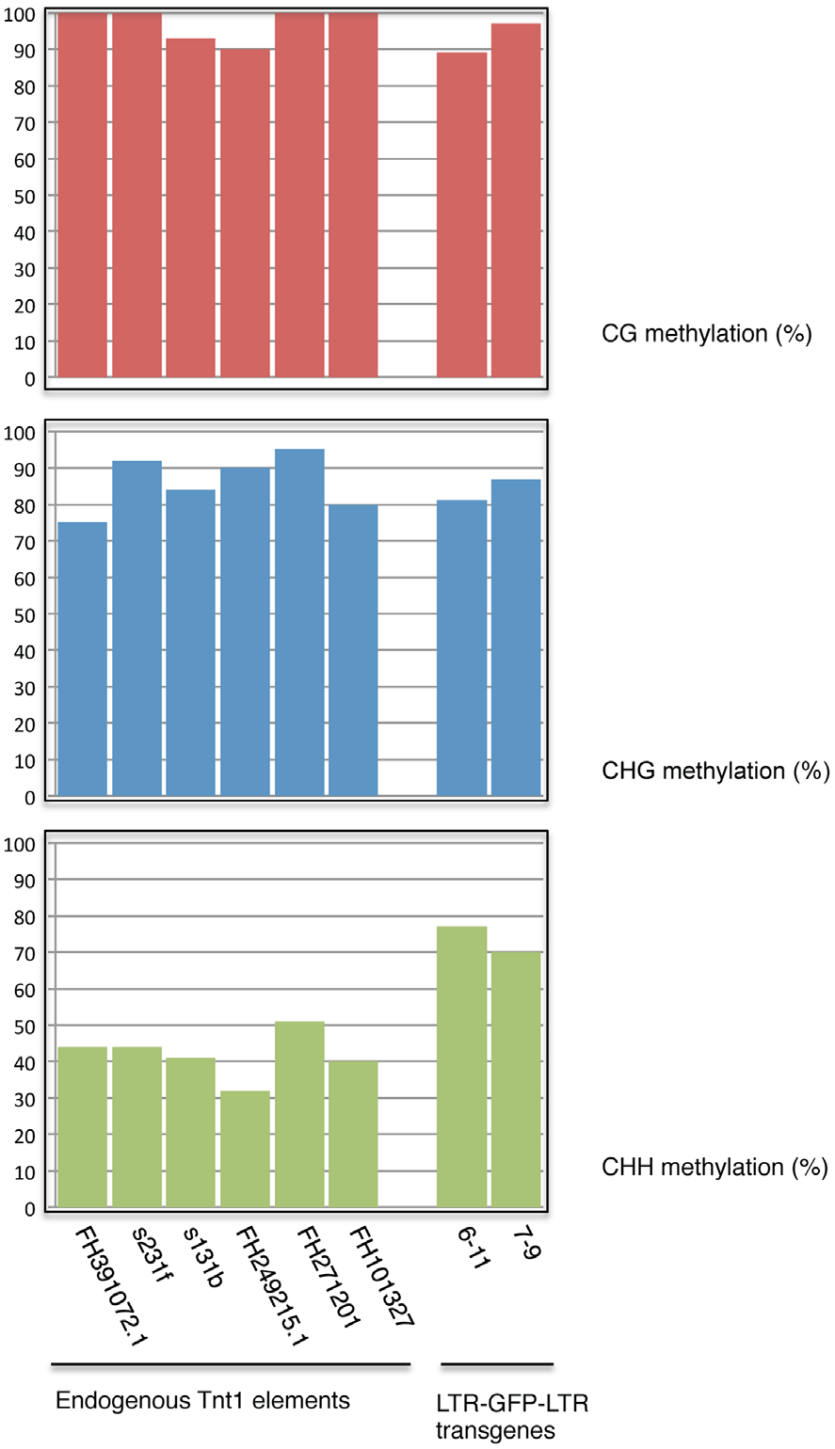
Comparison of the DNA methylation status of Tnt1 endogenous elements and LTR-GFP-LTR transgenes in R10-treated leaves. The percentage of methylated cytosines in each sequence contest was calculated for he sequences described in Figures 3 and 4.

Altogether these results show that the endogenous Tnt1 elements are significantly less methylated at asymmetrical positions than the silenced LTR-GFP-LTR transgenes (Figure 5), and suggest a correlation between high methylation at asymmetrical positions at the LTRs and silencing of the Tnt1 promoters.

### 4. Chromatin epigenetic marks associated with Tnt1 silencing

**Figure 6:**
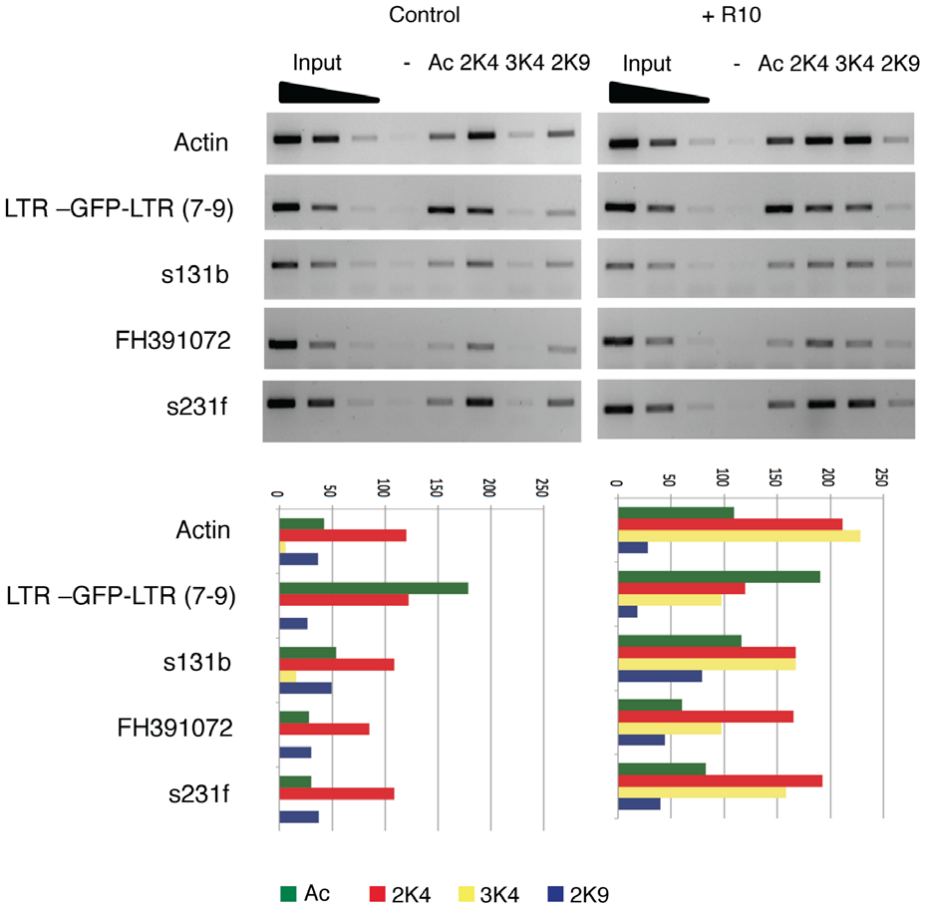
Histone epigenetic marks associated with the LTR-GFP-LTR transgene and endogenous Tnt1 elements. ChIP analyses were performed on leaves of the LTR-GFP-LTR transgenic line 7–9 non-treated (Control) or treated with R10 (R10) using antibodies recognizing H3 acetylation (Ac), H3K4me2 (2K4), H3K4me3 (3K4), and H3K9me2 (2K9), or no antibody (−). Different endogenous Tnt1 elements analyzed, as well as the LTR-GFP-LTR transgene. An actin gene fragment was used as control. A quantification of the band intensity relative to the second input dilution is shown below each panel.

In addition to DNA methylation, TGS is often correlated with the deposition of chromatin marks characteristic of inactive chromatin. We have therefore analyzed the histone modifications associated with the chromatin of the silenced LTR-GFP-LTR transgene as well as the endogenous Tnt1 elements. Both transgene and endogenous Tnt1 elements are associated with active chromatin epigenetic marks (AcH3, H3K4me2 and H3K4me3) both in normal and inductive stress conditions (Figure 6 and Figure S9). In fact, acetylation of histone H3 is even stronger at the silenced LTR-GFP-LTR transgene than at the active endogenous Tnt1 elements. On the contrary, heterochromatic marks such as H3K9me2 (Figure 6 and Figure S9) and H3K27me3 (not shown) are not present in either type of sequence. Upon treatment with R10, which activates transcription of endogenous Tnt1 elements, the promoters of both the endogenous Tnt1 elements and the silenced transgene are enriched in the H3K4me3 mark (Figure 6). H3K4me3 is an epigenetic mark associated with transcriptionally active genes and, in particular, with the activation of stress-regulated genes [42], [43]. However, this enrichment is not restricted to Tnt1 promoters and can also be observed in the actin control gene, suggesting a more general effect of the R10 associated stress.

In conclusion, although methylation at asymmetrical positions is higher at the silenced transgenes than at the active endogenous elements, this does not correlate with the presence of inactive chromatin epigenetic marks on the silenced transgene sequences.

**Figure 7:**
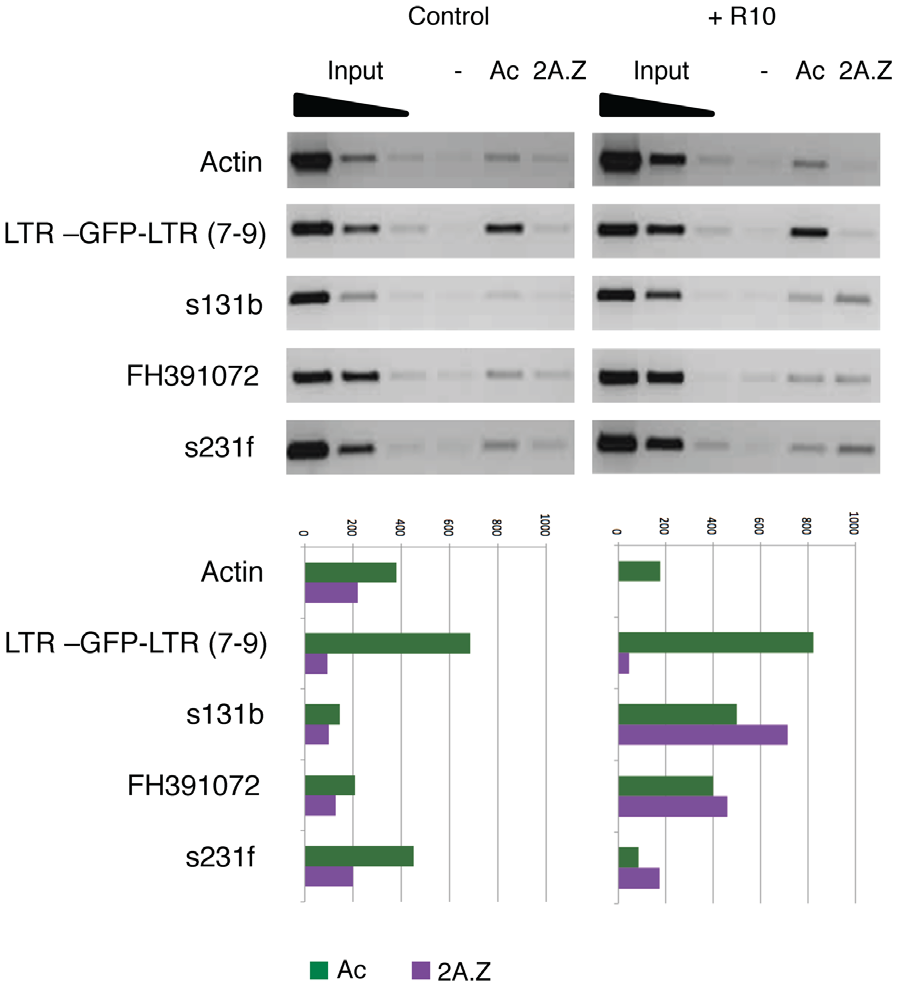
Association of the histone variant H2A.Z with the LTR-GFP-LTR transgene and endogenous Tnt1 elements. ChIP analyses were performed on leaves of the LTR-GFP-LTR transgenic line 7–9 non-treated (Control) or treated with R10 (R10) using antibodies recognizing H3 acetylation (Ac), H2A.Z (2A.Z), or no antibody (−). Different endogenous Tnt1 elements were analyzed, as well as the LTR-GFP-LTR transgene. An actin gene fragment was used as control are indicated. A quantification of the band intensity relative to the third input dilution is shown below each panel.

It has been recently shown that the incorporation of the histone variant H2A.Z into promoters correlates with their capacity to be activated [44], [45], [46]. It has also been shown that the presence of H2A.Z in chromatin usually correlates with the absence of DNA methylation [44], [47]. We consequently decided to check for the presence of H2A.Z associated with the promoter of Tnt1 in both the silenced transgene and the endogenous elements. Our results show that there is little or no H2A.Z associated with the promoters of either the transgene or the endogenous Tnt1 elements in tobacco leaves under non-inductive conditions (Figure 7). Interestingly, upon induction with R10, the endogenous Tnt1 elements, but not the silenced transgene, incorporate H2A.Z into their promoters (Figure 7).

The results presented here show that the promoter of Tnt1 is a target of silencing in tobacco and that, outside of the context of the entire Tnt1 element, silencing correlates with a higher level of methylation at asymmetric positions and the inability to incorporate the histone variant H2A.Z into the promoter region upon inductive treatments. Our results also show that endogenous Tnt1 elements, which remain transcriptionally active, are only partially methylated and incorporate H2A.Z into their promoters upon induction.

### 5. Analysis of the involvement of the internal Tnt1 sequences in its silencing protection

The main difference between the endogenous Tnt1 elements and the constructs containing LTR sequences that may explain their different sensitivity to silencing is that the transgenes lack most of the internal Tnt1 sequences. Indeed, although the LTR-GFP-LTR constructs have a structure resembling a Tnt1 element, with a central coding region flanked by two LTRs, they do not contain any of the Tnt1 coding regions. This strongly suggests that the central Tnt1 coding region is needed to confer methylation and silencing resistance. We have tried to precisely define the sequences that may provide silencing protection by analyzing the expression of LTR-driven transgenes containing also internal Tnt1 sequences. We analyzed first a construct driven by an extended Tnt1 LTR containing the region coding for the first 25 amino acids of the GAG protein (named Hut-2) that has already been shown to be transcriptionally active [33], [48]. We confirmed here that this construct is indeed expressed, and that its promoter is not heavily methylated (Figure S10). However, a new set of equivalent constructs consisting of the same extended promoter fused to the GFP gene, of which a high number of transgenic lines were analyzed, failed to consistently show a protective effect of this internal region (not shown). We then analyzed transgenic lines containing a construct named – END, which contains most of the Tnt1 internal sequences (a GFP coding sequence replacing a fragment of the endonuclease-coding region) (Figure S11A). 11 out of 36 lines containing the transgene stably integrated showed expression. The expression of the transgene did not correlate with the copy number (not shown). Although the expression of the stably introduced –END construct was higher than that of the LTR-GFP-LTR construct (11 out of 36 lines expressed the transgene whereas none of the 15 LTR-GFP-LTR lines gave detectable expression) (Figure S11B), this expression was not consistently maintained and was not always correlated with a lower level of methylation. In conclusion, whereas internal Tnt1 sequences probably play a role in protecting Tnt1 from being fully methylated and silenced, other factors may influence their expression.

## Discussion

While accounting for a major part of plant genomes, retrotransposons are very efficiently controlled by the silencing machinery of the cell and they are only expressed in very special situations. Apart from a transient de-repression in gametophytes and seeds, meant to reinforce their silencing in subsequent generations [40], in wild type plants most retrotransposons are only activated under severe stresses such as the stress associated with *in vitro* culture [10], [12], [24]. The relationship between retrotransposon activation and de-methylation shown in some cases [10], [11], as well as the general decrease in methylation observed under different stresses [30], lead to the suggestion that the silencing machinery may be reduced under stress conditions, allowing transposons to be activated [49]. However, our results show that the defense-related stress conditions that induce Tnt1 do not elicit a general decrease of TGS. Indeed, neither the stable nor the transient transcriptional silencing of a 35S promoter is relieved in these conditions. Tnt1 induction by defense-related stresses parallels that of different plant defense genes and is restricted to the cells surrounding the infection site (or the cells that are in contact with the infiltrated R10) [50]. The plant response to a local stress may be of a different type, and while some pathogen infections may lead in some cases to heterochromatin demethylation [51], the plant does not seem to deal with defense-related stresses by unspecific and extreme measures such as broadly reactivating its silenced sequences including transposons.

As a general release of silencing cannot be invoked to explain the expression of Tnt1, only two other possible explanations remain. Either Tnt1 is not a target of silencing in tobacco or, alternatively, it is but specifically escapes this control. Tobacco produces siRNAs of 24 nt against Tnt1, and we show here that they preferentially target the LTRs of the element suggesting that Tnt1 is regulated by siRNA-related silencing. Moreover, the results presented here show that constructs containing Tnt1 LTR sequences are strongly silenced and the Tnt1-related sequences are specifically methylated when stably introduced in tobacco, showing that tobacco is able to silence the Tnt1 promoter. The silencing of Tnt1 is highly efficient, as a single Tnt1 LTR (or fragments of it) added to the pool of hundreds of endogenous LTRs is detected, methylated and silenced. Interestingly, while Tnt1 sequences that are part of a transgene construct are methylated and silenced, endogenous Tnt1 elements escape this control remaining inducible and less methylated at asymmetrical positions.

The silencing of Tnt1 LTRs present within the transgene is not accompanied by repressive histone epigenetic marks. Indeed, the silenced LTR transgenes show high histone H3 acetylation, are associated to H3K4me2 and H3K4me3, and lack H3K9me2 and H3K27me3. It has been shown that although transposons are known to be one of the main targets of RNA-directed DNA methylation (RdDM) in plants [7], [40] their methylation is not always correlated with repressive histone epigenetic marks. Indeed, in *Arabidopsis* only the targets of the RdDM located in heterochromatic regions present also high levels of H3K9me2, whereas targets located in euchromatin are often associated with active histone epigenetic marks, such as histone H3 acetylation and H3K4me3, and lack H3K9me2, and it has been proposed that their silencing could be more easily reverted [7]. Therefore, the fact that Tnt1 promoter silencing does not invoke the deposition of inactive epigenetic marks may facilitate endogenous Tnt1's escape from silencing control.

The main difference between the endogenous Tnt1 elements and the constructs containing LTR sequences that may explain their different sensitivity to silencing is that the transgenes lack most of the internal Tnt1 sequences, which suggests that the central Tnt1 coding region may be needed to confer methylation and silencing resistance. For this reason we have tried to precisely define the sequences that may provide silencing protection but failed. Although some of the constructs containing Tnt1 internal regions showed some expression, it was not consistently maintained. Our results suggest that whereas internal Tnt1 sequences probably play a role in protecting Tnt1 from being fully methylated and silenced, other factors influence their expression.

The chromosomal environment could be one of these factors. Although, Tnt1 expression analyses have shown that multiple Tnt1 elements (sitting at different genomic locations) are concomitantly expressed in tobacco [21], [41], and we have not seen any expression in the 15 independent LTR-GFP-LTR lines, which suggests that the chromosomal location *per se* does not explain the ability for a particular Tnt1 to be expressed, we do have seen a high variability of expression of the –END construct or the constructs driven by chimerical Tnt1-35S promoters. It is interesting to note that, while Tnt1 does not have strict target site specificity, it integrates in genic regions and most Tnt1 copies are located near genes [52]. This close association with transcriptionally active regions may endow the endogenous Tnt1 elements with a particular resistance to silencing, that transgenes, which integrate much more randomly in the genome, may not have.

Another factor that may influence the expression and explain the difference between endogenous Tnt1 elements and Tnt1-containing constructs could be related to the way they are integrated into the genome. Whereas transgenes integrate at the level of double strand breaks, usually repaired by non-homologous end joining (NHEJ), retrotransposons use their own integrase to catalyze their insertion. This may influence their different sensitivity to silencing mechanisms and make Tnt1 sequences introduced as a part of a transgene more prone to silencing than their endogenous counterparts. Finally, retrotransposon activation is probably a highly stochastic event, and not all potentially active Tnt1 promoters may be activated at once. Indeed, it has recently been shown that the retrotransposon release from silencing in decreased in DNA methylation (*ddm1*) mutants in *Arabidopsis* is highly stochastic, with different elements proliferating in different plants [13]. In summary, while our results point towards a major role of Tnt1 internal sequences in protecting Tnt1 promoter from being silenced, other factors may modulate the ability of Tnt1 to escape silencing.

In the last few years strong evidence has accumulated indicating that the incorporation of the histone variant H2A.Z into promoters contributes to promoter competence [44], [45], [46]. Our results show that the LTRs of the endogenous Tnt1 elements incorporate H2A.Z upon induction of Tnt1 expression. Even though in most cases the association of H2A.Z with its targets occurs prior to induction, it has recently been shown that in some cases H2A.Z incorporation only occurs upon induction [53]. It is interesting to note that the silenced LTR-containing transgenes do not show this enrichment in H2A.Z upon Tnt1 induction. Thus the inducible deposition of H2A.Z seems to mark the Tnt1 LTRs competent for transcription. Incorporation of H2A.Z into promoters has been shown to correlate with a low level of DNA methylation and it has been proposed that H2A.Z and DNA methylation are antagonistic chromatin marks [44], [47]. Here we show that the LTR sequences of the endogenous Tnt1 elements incorporate H2A.Z in inductive conditions and are maintained only partially methylated at asymmetrical positions while those of the silenced transgenes are fully methylated and do not incorporate H2A.Z in the same conditions. Although the trigger for H2A.Z incorporation into the endogenous Tnt1 elements remains to be determined, the ability to incorporate this histone variant may well be what protects the endogenous Tnt1 elements from being fully methylated and silenced.

As it has been previously stated, given the long history of interaction between transposons and their hosts, it would seem likely that transposons, like viruses, have evolved strategies to avoid epigenetic silencing [49]. Here we show the first example of a retrotransposon that, while being a target of silencing, can escape this control and be expressed in its natural host.

## Materials and Methods

### Plant Material and Growth Conditions

Wild type and transgenic plants of *Nicotiana tabacum* cv Xanthi and *Nicotiana tabacum* cv Samsun were grown in glasshouses under controlled conditions at 24°C with 60% humidity, on 16 hr/8 hr light/dark cycles.

### R10 cellulase treatment

Tobacco leaves were infiltrated with MS medium containing 0.5 mg/ml of R10 cellulase from Onozuka (Yakult Pharmaceutical, Japan) and harvested 3 hours after infiltration for RNA isolation.

### Transgene constructs, plant transformation and transient *in planta* expression

The LTR-GFP-LTR construct has been previously described [41]. Binary constructs were moved into *Agrobacterium tumefaciens* strain LBA4404 for agrobacterium-mediated transformation of *Nicotiana tabacum* (cv Xanthi) plants. Plants with a single copy of the transgene were selected for further analysis.

The sequence of the 35S promoter was assembled in sense and antisense orientation into a pHannibal suppression vector [54] in which the original 35S promoter of pHannibal was substituted by the promoter of the *Arabidopsis thaliana* ATHB2 gene [36]. To this end, the 35S promoter sequence was amplified using forward and reverse primers containing *Xho*1 and *Kpn*1 or *BamH*1 and *Cla*1 restriction sites respectively. Both PCR fragments were cloned into the respective sites of the modified pHannibal to create a 35S hairpin construct. The hairpin cassette was then released from pHannibal by digestion with Not1 and cloned into pEnt3C (Invitrogen) and subsequently incorporated into the gateway binary vector pMDC99. Binary construct was transformed into *Agrobacterium tumefaciens* strain LBA4404. Overnight cultures grown in presence of 20 µM acetosyringone were pelleted by centrifugation and cells resuspended in 10 µM MgCl_2_, 10 µM MES (pH 5.7) and 150 µM acetosyringone to an OD_600_ of 0.3. Upon 4 hr incubation at room temperature, *Agrobacterium* suspension was infiltrated into expanded tobacco leaves using a needleless syringe. Three days after infiltration, non-treated and R10 cellulase-treated leaves were harvested for RNA isolation.

For transient expression of the silencing suppressor, a 35S promoter-based construct expressing the helper component protease (Hc-Pro) of Tobacco Etch Virus (TEV) was used [39]. Delivery of the suppressor construct into tobacco leaves was performed via *Agrobacterium tumefaciens* C58C1 strain. Agroinfiltration was carried out as described above. Ttobacco leaves were treated with R10 three days after infiltration.

### RNA Analysis

Total RNA was isolated from tobacco leaves using guanidine hydrochloride as described [55]. For Northern blot analysis, between 10 and 20 µg of total RNA was loaded on a 1% agarose-formaldehyde gel, blotted to Hybond-N (Amersham) membranes by standard capillary transfer, and UV cross-linked. Probes corresponding to the entire GUS or GFP DNA were ^32^P-labelled by PCR. The Tnt1 probe was generated by PCR amplification of a 624 bp fragment (RT3-RT6 oligos) from the Tnt1 region coding for the reverse transcriptase. Membranes were hybridized overnight at 65°C in Church buffer [56].

### DNA Methylation Analysis

The DNA methylation status was analyzed by Southern hybridization and bisulfite sequencing. For Southern hybridization, 25 µg of genomic DNA was digested with HindIII in combination with the methylation sensitive restriction enzymes AluI, DdeI or HpaII. The digested DNA was loaded onto a 1% agarose gel, blotted to Hybond-N membranes by standard capillary transfer and UV cross-linked. Membranes were hybridized overnight at 65°C in Church buffer [56] using appropriate DNA probes. For bisulfite sequencing, 400 ng of genomic DNA was treated with sodium bisulfite using the EZ DNA Methylation-Gold kit (Zymo Research) according to the manufacturer's instructions. Treatment was performed in a PCR thermocycler starting with one cycle of 98°C for 10 min and 53°C for 30 min, followed with 8 cycles of 53°C for 6 min and 37°C for 30 min. The bisulfite-treated DNA was used for amplification of endogenous Tnt1 LTRs and transgene Tnt1-related promoter sequences with primers positioned at the respective franking regions. All PCR procedures were carried out for 42 cycles using AmpliTaq Gold DNA polymerase (Applied Biosystem). PCR products were cloned into pCRII using the TOPO TA kit (Invitrogen), and at least 10 individual clones were sequenced for each sample. All primers used for methylation analysis are listed in Table S1. The analysis of the obtained sequences was performed using the Kismeth program [57].

### Chromatin Immunoprecipitation Assay

Transgenic tobacco leaves were cross-linked with 1% formaldehyde in 10 mM Tris-HCl pH 8.0, 0.4 M sucrose, 1 mM EDTA and 0.5% Triton-X100. Nuclei were isolated in extraction buffer (10 mM Tris-HCl pH 8.0, 0.25 M sucrose, 10 mM Mg Cl_2_, 1% Triton-X100, 1 mM DTT), resuspended in lysis buffer (15 mM Tris-HCl pH 7.5, 60 mM KCl, 2 mM EDTA, 1 mM DTT) containing 0.1% SDS and sonicated in a Bioruptor. The DNA/protein complexes were immunoprecipitated with α-H3Ac (Upstate 06-599), α-dimethyl H3K4 (Upstate 07-030), α-trimethyl H3K4 (Abcam 8580), α-dimethyl H3K9 (Upstate 07-441) or α-H2AZ (Abcam 18263) as previously described [58] except that, after reversion of formaldehyde cross-links, DNA was extracted with phenol/chloroform and ethanol precipitated. The U3 region of the endogenous Tnt1 and transgene promoters was amplified by standard procedures using the primers detailed in Table S1.

## Supporting Information

Figure S1 **Transient transcriptional gene silencing of the 35S promoter.** A) Northern blot analysis of GUS expression in leaves of a 35S-GUS transgenic plant, after infiltration with a 35S hairpin expression construct. Different lanes correspond to different number of days after infiltration (DAI). The expression of an endogenous ubiquitin gene is shown beneath as control. B) Southern analysis of DNA from the same tobacco leaves analyzed in (A) digested with methylation sensitive enzymes to assess DNA methylation. The enzymes used are indicated on the left of each panel. A schema showing the position of the restriction enzymes sites, the probe used (black box) and the expected fragments is shown below. The 35S promoter is shown as a grey box and the GUS coding sequence is shown as a blue box. C) Northern blot analysis of GUS expression in 35S-GUS transgenic plants non silenced (−), silenced by crossing with the 271 locus (×271) or by infiltration with a 35S hairpin expression construct (ds35S). D) Southern blot analysis of DNA from the same tobacco leaves analyzed in (C) digested with methylation sensitive enzymes to assess DNA methylation. The expected bands are the same as in (B).
(PDF)

Figure S2 **Tobacco siRNAs targeting the Tnt1 sequence.** A) size distribution of tobacco siRNAs from leaves (obtained from the public database http://smallrna.udel.edu/) which target the Tnt1 sequence. B) Distribution of the siRNAs of 24 nt directed against Tnt1 along the Tnt1 sequence.
(PDF)

Figure S3 **Analysis of the potential post-transcriptional silencing of the LTR-GFP-LTR constructs.** Northern analysis of tobacco leaves of two different transgenic lines containing a single copy of the LTR-GFP-LTR transgene treated (+) or non treated (−) with R10. Half of the leaves were infiltrated with a construct expressing the viral silencing suppressor HcPro (H) three days prior to R10 treatment. A transgenic line expressing a 35S-GFP was included as an hybridization control. An image of the EtBr staining of the RNA gel used as loading control is shown underneath.
(PDF)

Figure S4 **Silencing of chimeric promoters containing part of the Tnt1 LTR.** Qualitative expression of 5 different constructs driven by promoters containing Tnt1 LTR fragments, stably integrated in tobacco. The expression of a control 35S driven construct is shown as control. A schema of the analyzed construct and the name of the construct is given above each set of results. Tnt1 LTR sequences are shown as red boxes and 35S sequences are shown as yellow boxes. Constructs contain: a complete 35S promoter (35SG); a complete U3 sequence of a Tnt1 element (U3AG); the upstream U3 sequence, up to the TATA box, of Tnt1 plus the −90 region of a 35S, including the TATA box and the transcriptional start (A90G); the enhancer region of the 35S promoter and the TATA box and transcriptional start region of a Tnt1 U3 (ETAG); the enhancer region of the 35S promoter upstream of a complete U3 region of Tnt1 (EAG), and the enhancer region of the 35S promoter upstream of a A90G construct (EE90G). For each construct the expression in untreated leaves and leaves treated with R10 (R10) in different lines containing a single copy of the transgene was analyzed. The expected expression (−, no expression; +, expression) of each construct based on the knowledge of the 35S and Tnt1 promoter is shown in red. The actual expression of the different lines for each construct is shown in black. Very low expression is shown by a small + sign. For some of the constructs different individual plants of the same line were analyzed to assess individual variability of expression.
(PDF)

Figure S5 **DNA Methylation status of the 3′LTR of the LTR-GFP-LTR silenced transgene.** The 3′ region of the LTR-GFP-LTR transgene, including the 3′ LTR, was amplified and sequenced from bisulfite converted DNA from R10-treated leaves of the LTR-GFP-LTR 6–11 transgenic line. Ten clones were sequenced from each transgene (only one sequence is shown when the same sequence was obtained several times). The different regions of the transgene are shown under the sequence.The methylation state of each cytosine is shown as in Figures 3 and 4.
(PDF)

Figure S6 **Comparison of the LTR sequences of endogenous Tnt1 elements together with Tnt1 RNA LTR sequences.** Phylogenetic analysis of 157 Tnt1 LTRs present in public databases (shown as red dots) with 25 Tnt1 mRNA sequences obtained from R10 induced leaves (shown as green dots). LTRs selected for further analysis are shown by a red arrow.
(PDF)

Figure S7 **DNA Methylation status of the 3′LTR of an endogenous Tnt1 element.** The 3′ region of the s231f endogenous Tnt1 element, including the 3′ LTR, was amplified and sequenced from bisulfite converted DNA from R10-treated leaves of the LTR-GFP-LTR 6-11 transgenic line. Ten clones were sequenced from each transgene (only one sequence is shown when the same sequence was obtained several times). The methylation state of each cytosine is shown as in Figures 3 and 4.The different regions of the Tnt1 element are shown under the sequence.
(PDF)

Figure S8 **Global methylation analysis of the endogenous Tnt1 population.** Southern blot analysis of tobacco DNA obtained from leaves untreated (0) or treated with R10 for different periods of time (0.5, 2 or 6 hours) digested with enzymes sensitive to asymmetrical (HindIII and some AluI sites, shown by a green dot) and symmetrical (HpaII and some AluI sites, shown by a red dot) methylation. A schema of the position of the probe used for hybridization and the expected band sizes are shown below. The approximate sizes of the hybridizing bands is shown.
(PDF)

Figure S9 **Histone epigenetic marks associated with the LTR-GFP-LTR transgene and endogenous Tnt1 elements.** ChIP analyses were performed with the LTR-GFP-LTR transgenic line 6–11 non treated (Control) or treated with R10 (R10) using antibodies recognizing H3 acetylation (Ac), H3K4me2 (2K4), and H3K9me2 (2K9), or no antibody (−). Different endogenous Tnt1 elements tested for, as well as the LTR-GFP-LTR transgene and an actin gene fragment used as control. A quantification of the band intensity relative to the second input dilution is shown below each panel.
(PDF)

Figure S10 **The Hut-2 transgene is not silenced when stably introduced in tobacco.** (A) Schema of the Hut-2 transgene. A schema of the Tnt1 element is given for comparison. B) Northen blot analysis of the expression of the Hut-2 transgene in leaves of two independent transgenic lines, non-treated (−) or treated (+) with R10. The name of the line is given on top. The hybridization with a Tnt1 probe and an image of the EtBr staining of the RNA gel are shown underneath as controls. C) Methylation analysis of the Hut-2 transgene. The 5′ region of the Hut-2 transgene, including the 5′ LTR, was amplified and sequenced from bisulfite converted DNA from R10-treated leaves. At least 10 clones were sequenced from each transgene (only one sequence is shown when the same sequence was obtained several times). The methylation state of each cytosine is shown as in Figures 3 and 4. The different regions of the transgene are shown under the sequence.
(PDF)

Figure S11 **Constructs containing most of the internal sequence of Tnt1 can be expressed in tobacco transgenic plants.** (A) Schema of the – END transgene. A schema of the Tnt1 element is given for comparison. B) Northern analysis of GFP expression in wild type plants agroinfiltrated with the –END construct and expressing it transiently (trans.), as well as in plants stably transformed with the same construct (the number of the transgenic line is indicated on the top). RNA was extracted from leaves treated (+) and non-treated (−) with R10, and probed with a GFP probe. An image of the EtBr staining of the RNA gel is shown underneath as loading control. The figure presents the analysis of 7 lines representative of the 36 obtained.
(PDF)

Table S1 **Primer pairs.**
(PDF)
