# Supplementary material for: The Tnt1 Retrotransposon Escapes Silencing in Tobacco, Its Natural Host
Source: PLoS One. 2012 Mar 30;7(3):e33816. doi: 10.1371/journal.pone.0033816 (PMC3316501; doi:10.1371/journal.pone.0033816)
Supplement: Figure S1 — Transient transcriptional gene silencing of the 35S promoter. A) Northern blot analysis of GUS expression in leaves of a 35S-GUS transgenic plant, after infiltration with a 35S hairpin expression construct. Different lanes correspond to different number of days after infiltration (DAI). The expression of an endogenous ubiquitin gene is shown beneath as control. B) Southern analysis of DNA from the same tobacco leaves analyzed in (A) digested with methylation sensitive enzymes to assess DNA methylation. The enzymes used are indicated on the left of each panel. A schema showing the position of the restriction enzymes sites, the probe used (black box) and the expected fragments is shown below. The 35S promoter is shown as a grey box and the GUS coding sequence is shown as a blue box. C) Northern blot analysis of GUS expression in 35S-GUS transgenic plants non silenced (−), silenced by crossing with the 271 locus (×271) or by infiltration with a 35S hairpin expression construct (ds35S). D) Southern blot analysis of DNA from the same tobacco leaves analyzed in (C) digested with methylation sensitive enzymes to assess DNA methylation. The expected bands are the same as in (B). (PDF) [file pone.0033816.s001.pdf]

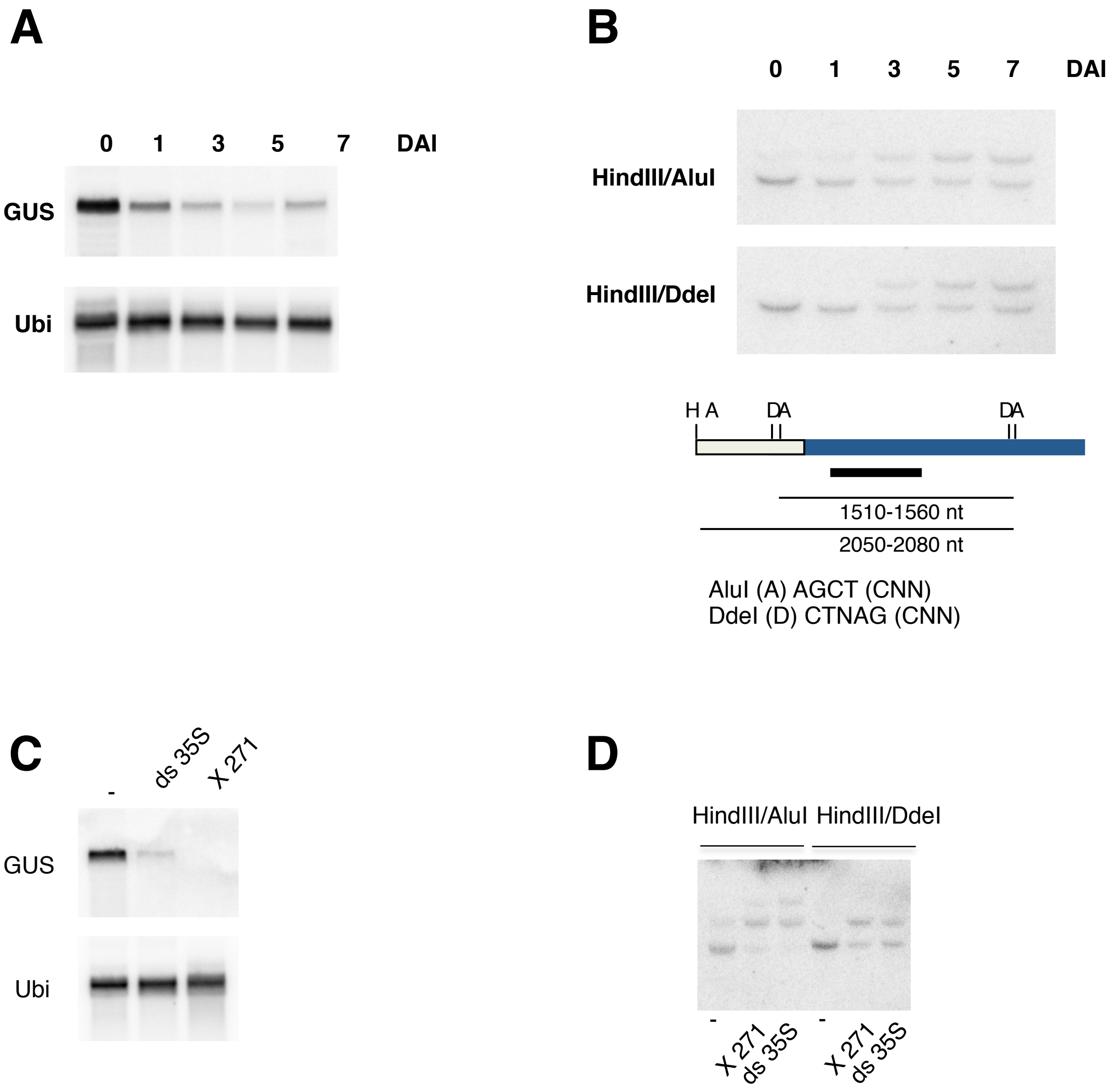

Supporting Figure S1. Transient transcriptional gene silencing of the 35S promoter.
A) Northern blot analysis of GUS expression in leaves of a 35S-GUS transgenic plant, after infiltration with a 35S hairpin expression construct. Different lanes correspond to different number of days after infiltration (DAI). The expression of an endogenous ubiquitin gene is shown beneath as control. B) Southern analysis of DNA from the same tobacco leaves analyzed in (A) digested with methylation sensitive enzymes to assess DNA methylation. The enzymes used are indicated on the left of each panel. A schema showing the position of the restriction enzymes sites, the probe used (black box) and the expected fragments is shown below. The 35S promoter is shown as a grey box and the GUS coding sequence is shown as a blue box. C) Northern blot analysis of GUS expression in 35S-GUS transgenic plants non silenced (-), silenced by crossing with the 271 locus (x 271) or by infiltration with a 35S hairpin expression construct (ds35S). D) Southern blot analysis of DNA from the same tobacco leaves analyzed in (C) digested with methylation sensitive enzymes to assess DNA methylation. The expected bands are the same as in (B).
