# Supplementary material for: The Tnt1 Retrotransposon Escapes Silencing in Tobacco, Its Natural Host
Source: PLoS One. 2012 Mar 30;7(3):e33816. doi: 10.1371/journal.pone.0033816 (PMC3316501; doi:10.1371/journal.pone.0033816)
Supplement: Figure S2 — Tobacco siRNAs targeting the Tnt1 sequence. A) size distribution of tobacco siRNAs from leaves (obtained from the public database http://smallrna.udel.edu/) which target the Tnt1 sequence. B) Distribution of the siRNAs of 24 nt directed against Tnt1 along the Tnt1 sequence. (PDF) [file pone.0033816.s002.pdf]

A

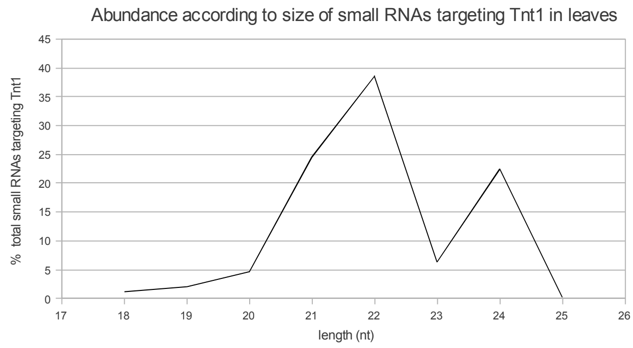

B

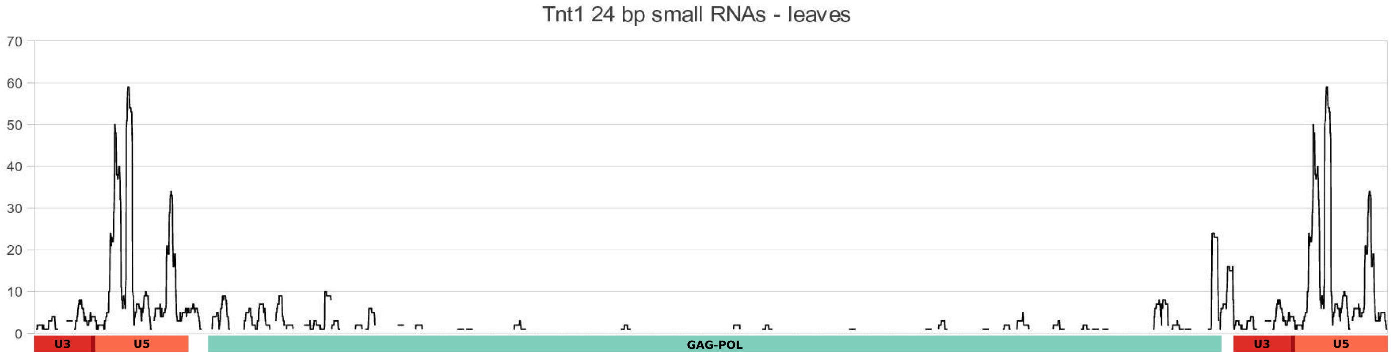

Supporting Figigure S2. Tobacco siRNAs targeting the Tnt1 sequence. A) size distribution of tobacco siRNAs from leaves (obtained from the public database http://smallrna.udel.edu/) which target the Tnt1 sequence. B) Distribution of the siRNAs of 24 nt directed against Tnt1 along the Tnt1 sequence.
