# Supplementary material for: The Tnt1 Retrotransposon Escapes Silencing in Tobacco, Its Natural Host
Source: PLoS One. 2012 Mar 30;7(3):e33816. doi: 10.1371/journal.pone.0033816 (PMC3316501; doi:10.1371/journal.pone.0033816)
Supplement: Figure S3 — Analysis of the potential post-transcriptional silencing of the LTR-GFP-LTR constructs. Northern analysis of tobacco leaves of two different transgenic lines containing a single copy of the LTR-GFP-LTR transgene treated (+) or non treated (−) with R10. Half of the leaves were infiltrated with a construct expressing the viral silencing suppressor HcPro (H) three days prior to R10 treatment. A transgenic line expressing a 35S-GFP was included as an hybridization control. An image of the EtBr staining of the RNA gel used as loading control is shown underneath. (PDF) [file pone.0033816.s003.pdf]

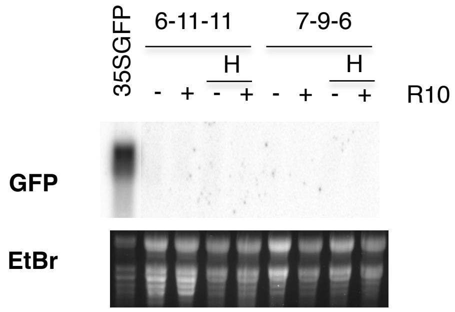


**Supporting Figure S3. Analysis of the potential post-transcriptional silencing of the LTR-GFP-LTR constructs.** Northern analysis of tobacco leaves of two different transgenic lines containing a single copy of the LTR-GFP-LTR transgene treated (+) or non treated (-) with R10. Half of the leaves were infiltrated with a construct expressing the viral silencing suppressor HcPro (H) three days prior to R10 treatment. A transgenic line expressing a 35S-GFP was included as an hybridization control. An image of the EtBr staining of the RNA gel used as loading control is shown underneath.
