# Supplementary material for: The Tnt1 Retrotransposon Escapes Silencing in Tobacco, Its Natural Host
Source: PLoS One. 2012 Mar 30;7(3):e33816. doi: 10.1371/journal.pone.0033816 (PMC3316501; doi:10.1371/journal.pone.0033816)
Supplement: Figure S4 — Silencing of chimeric promoters containing part of the Tnt1 LTR. Qualitative expression of 5 different constructs driven by promoters containing Tnt1 LTR fragments, stably integrated in tobacco. The expression of a control 35S driven construct is shown as control. A schema of the analyzed construct and the name of the construct is given above each set of results. Tnt1 LTR sequences are shown as red boxes and 35S sequences are shown as yellow boxes. Constructs contain: a complete 35S promoter (35SG); a complete U3 sequence of a Tnt1 element (U3AG); the upstream U3 sequence, up to the TATA box, of Tnt1 plus the −90 region of a 35S, including the TATA box and the transcriptional start (A90G); the enhancer region of the 35S promoter and the TATA box and transcriptional start region of a Tnt1 U3 (ETAG); the enhancer region of the 35S promoter upstream of a complete U3 region of Tnt1 (EAG), and the enhancer region of the 35S promoter upstream of a A90G construct (EE90G). For each construct the expression in untreated leaves and leaves treated with R10 (R10) in different lines containing a single copy of the transgene was analyzed. The expected expression (−, no expression; +, expression) of each construct based on the knowledge of the 35S and Tnt1 promoter is shown in red. The actual expression of the different lines for each construct is shown in black. Very low expression is shown by a small + sign. For some of the constructs different individual plants of the same line were analyzed to assess individual variability of expression. (PDF) [file pone.0033816.s004.pdf]

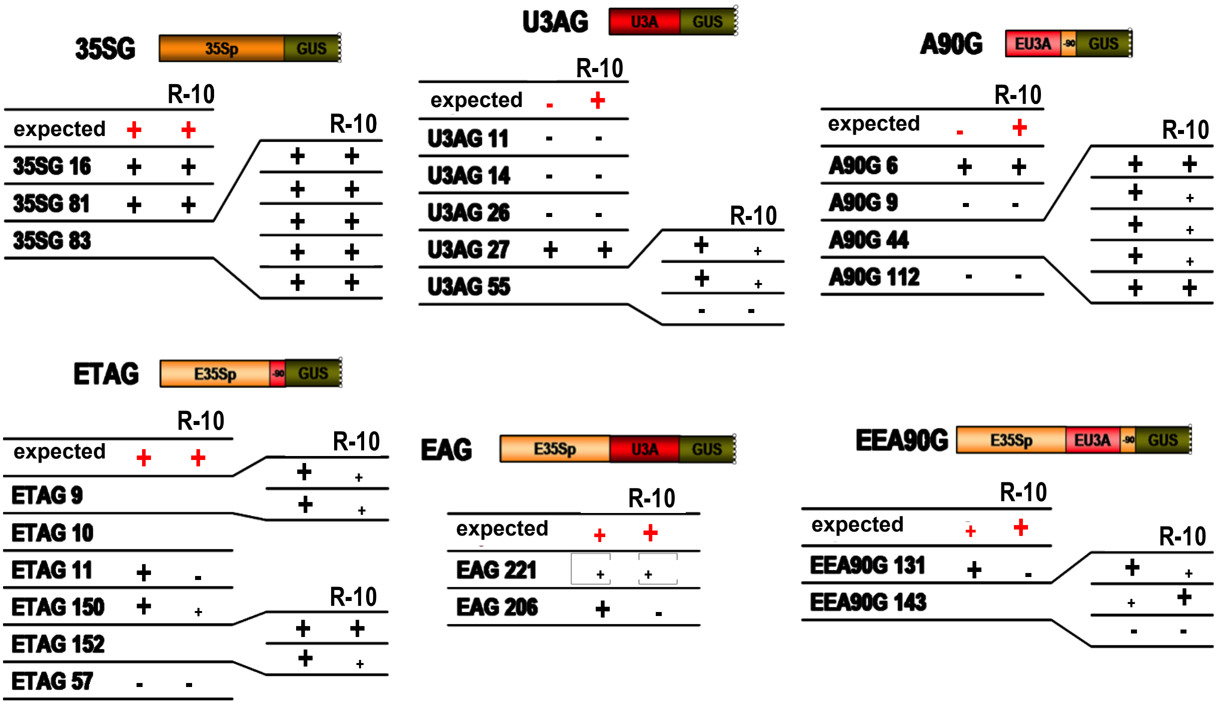

Supporting Figure S4. Silencing of chimeric promoters containing part of the Tnt1 LTR. Qualitative expression of 5 different constructs driven by promoters containing Tnt1 LTR fragments, stably integrated in tobacco. The expression of a control 35S driven construct is shown as control. A schema of the analyzed construct and the name of the construct is given above each set of results. Tnt1 LTR sequences are shown as red boxes and 35S sequences are shown as yellow boxes. Constructs contain: a complete 35S promoter (35SG); a complete U3 sequence of a Tnt1 element (U3AG); the upstream U3 sequence, up to the TATA box, of Tnt1 plus the -90 region of a 35S, including the TATA box and the transcriptional start (A90G); the enhancer region of the 35S promoter and the TATA box and transcriptional start region of a Tnt1 U3 (ETAG); the enhancer region of the 35S promoter upstream of a complete U3 region of Tnt1 (EAG), and the enhancer region of the 35S promoter upstream of a A90G construct (EE90G). For each construct the expression in untreated leaves and leaves treated with R10 (R10) in different lines containing a single copy of the transgene was analyzed. The expected expression (-, no expression; +, expression) of each construct based on the knowledge of the 35S and Tnt1 promoter is shown in red. The actual expression of the different lines for each construct is shown in black. Very low expression is shown by a small + sign. For some of the constructs different individual plants of the same line were analyzed to assess individual variability of expression.
