# Supplementary material for: The Tnt1 Retrotransposon Escapes Silencing in Tobacco, Its Natural Host
Source: PLoS One. 2012 Mar 30;7(3):e33816. doi: 10.1371/journal.pone.0033816 (PMC3316501; doi:10.1371/journal.pone.0033816)
Supplement: Figure S5 — DNA Methylation status of the 3′LTR of the LTR-GFP-LTR silenced transgene. The 3′ region of the LTR-GFP-LTR transgene, including the 3′ LTR, was amplified and sequenced from bisulfite converted DNA from R10-treated leaves of the LTR-GFP-LTR 6–11 transgenic line. Ten clones were sequenced from each transgene (only one sequence is shown when the same sequence was obtained several times). The different regions of the transgene are shown under the sequence.The methylation state of each cytosine is shown as in Figures 3 and 4. (PDF) [file pone.0033816.s005.pdf]

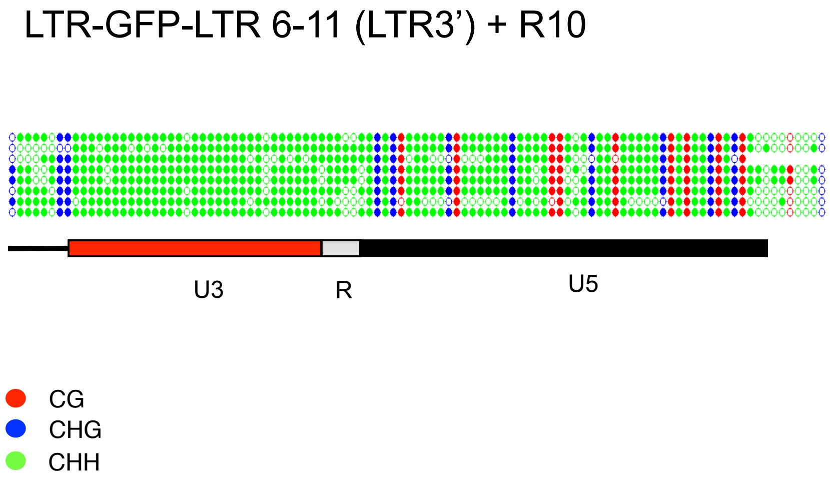

Supporting Figure S5. DNA Methylation status of the 3'LTR of the LTR-GFP-LTR silenced transgene. The 3' region of the LTR-GFP-LTR transgene, including the 3' LTR, was amplified and sequenced from bisulfite converted DNA from R10-treated leaves of the LTR-GFP-LTR 6-11 transgenic line. Ten clones were sequenced from each transgene (only one sequence is shown when the same sequence was obtained several times). The different regions of the transgene are shown under the sequence.The methylation state of each cytosine is shown as in Figures 3 and 4.
