# Supplementary material for: The Tnt1 Retrotransposon Escapes Silencing in Tobacco, Its Natural Host
Source: PLoS One. 2012 Mar 30;7(3):e33816. doi: 10.1371/journal.pone.0033816 (PMC3316501; doi:10.1371/journal.pone.0033816)
Supplement: Figure S6 — Comparison of the LTR sequences of endogenous Tnt1 elements together with Tnt1 RNA LTR sequences. Phylogenetic analysis of 157 Tnt1 LTRs present in public databases (shown as red dots) with 25 Tnt1 mRNA sequences obtained from R10 induced leaves (shown as green dots). LTRs selected for further analysis are shown by a red arrow. (PDF) [file pone.0033816.s006.pdf]

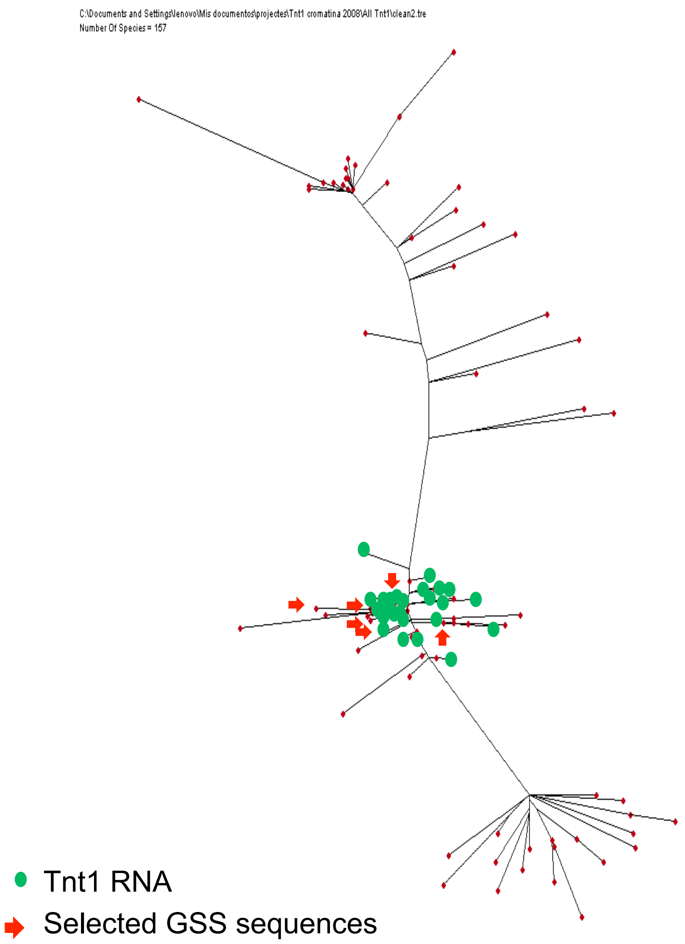

Supporting Figure S6. Comparison of the LTR sequences of endogenous Tnt1 elements together with Tnt1 RNA LTR sequences. Phylogenetic analysis of 157 Tnt1 LTRs present in public databases (shown as red dots) with 25 Tnt1 mRNA sequences obtained from R10 induced leaves (shown as green dots). LTRs selected for further analysis are shown by a red arrow.
