# Supplementary material for: The Tnt1 Retrotransposon Escapes Silencing in Tobacco, Its Natural Host
Source: PLoS One. 2012 Mar 30;7(3):e33816. doi: 10.1371/journal.pone.0033816 (PMC3316501; doi:10.1371/journal.pone.0033816)
Supplement: Figure S7 — DNA Methylation status of the 3′LTR of an endogenous Tnt1 element. The 3′ region of the s231f endogenous Tnt1 element, including the 3′ LTR, was amplified and sequenced from bisulfite converted DNA from R10-treated leaves of the LTR-GFP-LTR 6-11 transgenic line. Ten clones were sequenced from each transgene (only one sequence is shown when the same sequence was obtained several times). The methylation state of each cytosine is shown as in Figures 3 and 4.The different regions of the Tnt1 element are shown under the sequence. (PDF) [file pone.0033816.s007.pdf]

S231f (LTR 3') + R10

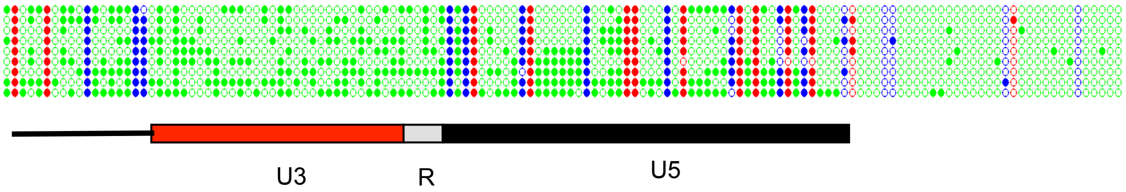

Supporting Figure S7. DNA Methylation status of the 3'LTR of an endogenous Tnt1 element. The 3' region of the s231f endogenous Tnt1 element, including the 3' LTR, was amplified and sequenced from bisulfite converted DNA from R10-treated leaves of the LTR-GFP-LTR 6-11 transgenic line. Ten clones were sequenced from each transgene (only one sequence is shown when the same sequence was obtained several times). The methylation state of each cytosine is shown as in Figures 3 and 4.The different regions of the Tnt1 element are shown under the sequence.
