# Supplementary material for: The Tnt1 Retrotransposon Escapes Silencing in Tobacco, Its Natural Host
Source: PLoS One. 2012 Mar 30;7(3):e33816. doi: 10.1371/journal.pone.0033816 (PMC3316501; doi:10.1371/journal.pone.0033816)
Supplement: Figure S8 — Global methylation analysis of the endogenous Tnt1 population. Southern blot analysis of tobacco DNA obtained from leaves untreated (0) or treated with R10 for different periods of time (0.5, 2 or 6 hours) digested with enzymes sensitive to asymmetrical (HindIII and some AluI sites, shown by a green dot) and symmetrical (HpaII and some AluI sites, shown by a red dot) methylation. A schema of the position of the probe used for hybridization and the expected band sizes are shown below. The approximate sizes of the hybridizing bands is shown. (PDF) [file pone.0033816.s008.pdf]

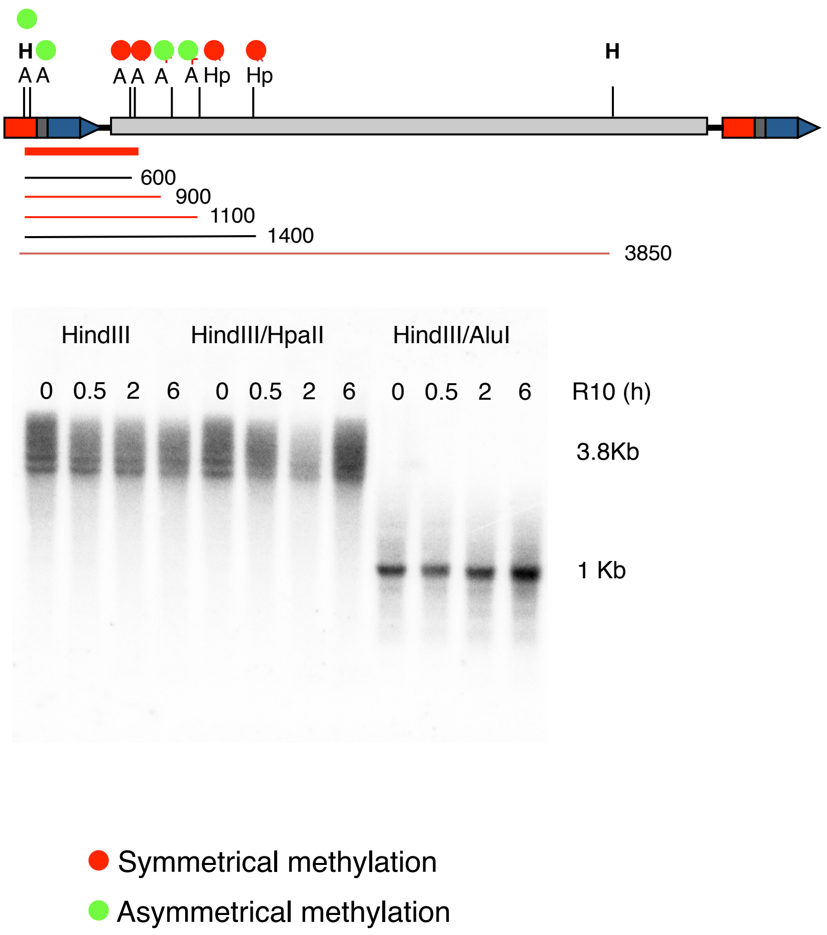

Supporting Figure S8. Global methylation analysis of the endogenous Tnt1 population. Southern blot analysis of tobacco DNA obtained from leaves untreated (0) or treated with R10 for different periods of time (0.5, 2 or 6 hours) digested with enzymes sensitive to asymmetrical (HindIII and some AluI sites, shown by a green dot) and symmetrical (HpaII and some AluI sites, shown by a red dot) methylation. A schema of the position of the probe used for hybridization and the expected band sizes are shown below. The approximate sizes of the hybridizing bands is shown.
