# Supplementary material for: The Tnt1 Retrotransposon Escapes Silencing in Tobacco, Its Natural Host
Source: PLoS One. 2012 Mar 30;7(3):e33816. doi: 10.1371/journal.pone.0033816 (PMC3316501; doi:10.1371/journal.pone.0033816)
Supplement: Figure S9 — Histone epigenetic marks associated with the LTR-GFP-LTR transgene and endogenous Tnt1 elements. ChIP analyses were performed with the LTR-GFP-LTR transgenic line 6–11 non treated (Control) or treated with R10 (R10) using antibodies recognizing H3 acetylation (Ac), H3K4me2 (2K4), and H3K9me2 (2K9), or no antibody (−). Different endogenous Tnt1 elements tested for, as well as the LTR-GFP-LTR transgene and an actin gene fragment used as control. A quantification of the band intensity relative to the second input dilution is shown below each panel. (PDF) [file pone.0033816.s009.pdf]

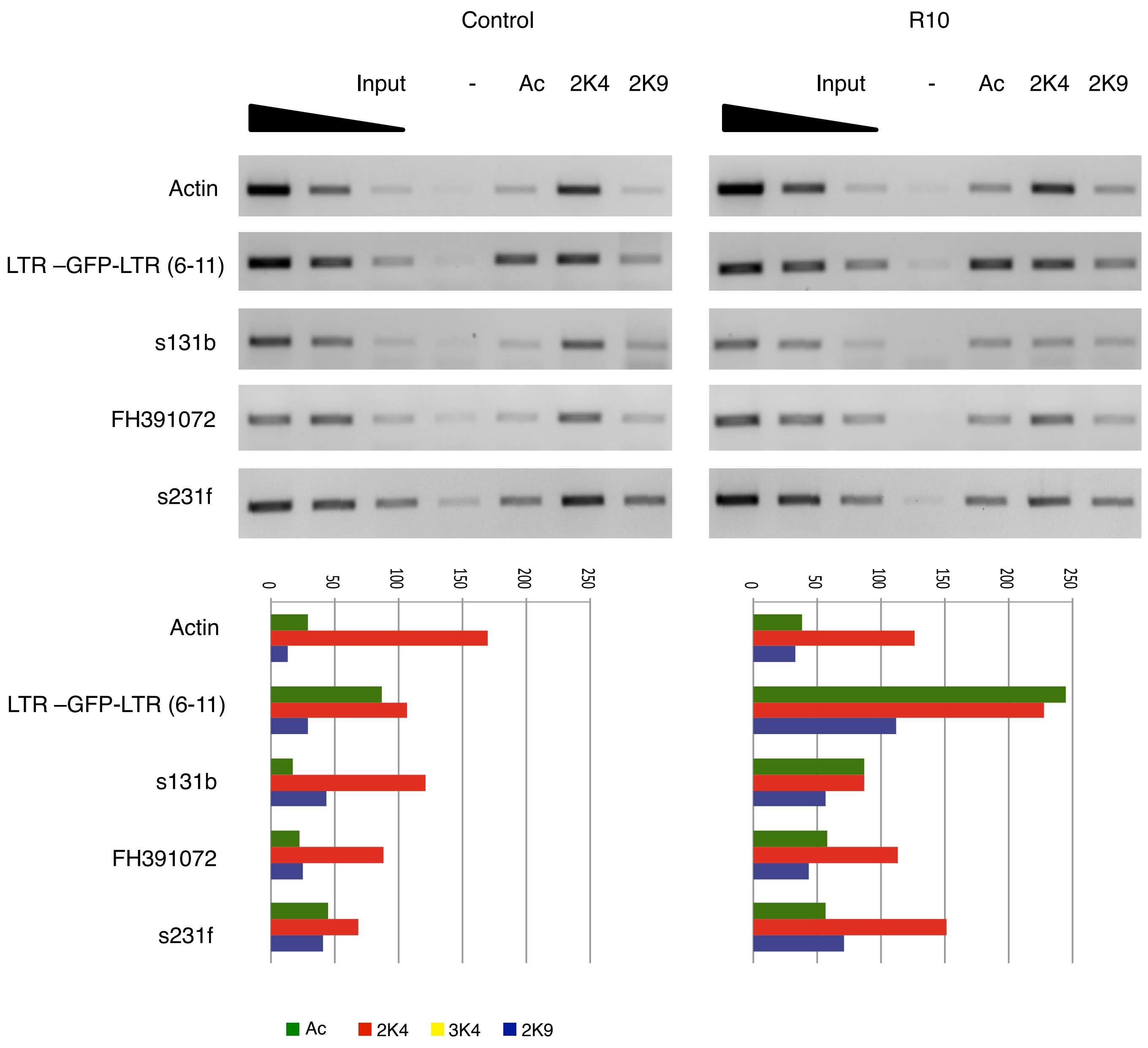

Supporting Figure S9. Histone epigenetic marks associated with the LTR-GFP-LTR transgene and endogenous Tnt1 elements. ChIP analyses were performed with the LTR-GFP-LTR transgenic line 6-11 non treated (Control) or treated with R10 (R10) using antibodies recognizing H3 acetylation (Ac), H3K4me2 (2K4), and H3K9me2 (2K9), or no antibody (-). Different endogenous Tnt1 elements tested for, as well as the LTR-GFP-LTR transgene and an actin gene fragment used as control. A quantification of the band intensity relative to the second input dilution is shown below each panel.
