# Supplementary material for: The Tnt1 Retrotransposon Escapes Silencing in Tobacco, Its Natural Host
Source: PLoS One. 2012 Mar 30;7(3):e33816. doi: 10.1371/journal.pone.0033816 (PMC3316501; doi:10.1371/journal.pone.0033816)
Supplement: Figure S10 — The Hut-2 transgene is not silenced when stably introduced in tobacco. (A) Schema of the Hut-2 transgene. A schema of the Tnt1 element is given for comparison. B) Northen blot analysis of the expression of the Hut-2 transgene in leaves of two independent transgenic lines, non-treated (−) or treated (+) with R10. The name of the line is given on top. The hybridization with a Tnt1 probe and an image of the EtBr staining of the RNA gel are shown underneath as controls. C) Methylation analysis of the Hut-2 transgene. The 5′ region of the Hut-2 transgene, including the 5′ LTR, was amplified and sequenced from bisulfite converted DNA from R10-treated leaves. At least 10 clones were sequenced from each transgene (only one sequence is shown when the same sequence was obtained several times). The methylation state of each cytosine is shown as in Figures 3 and 4. The different regions of the transgene are shown under the sequence. (PDF) [file pone.0033816.s010.pdf]

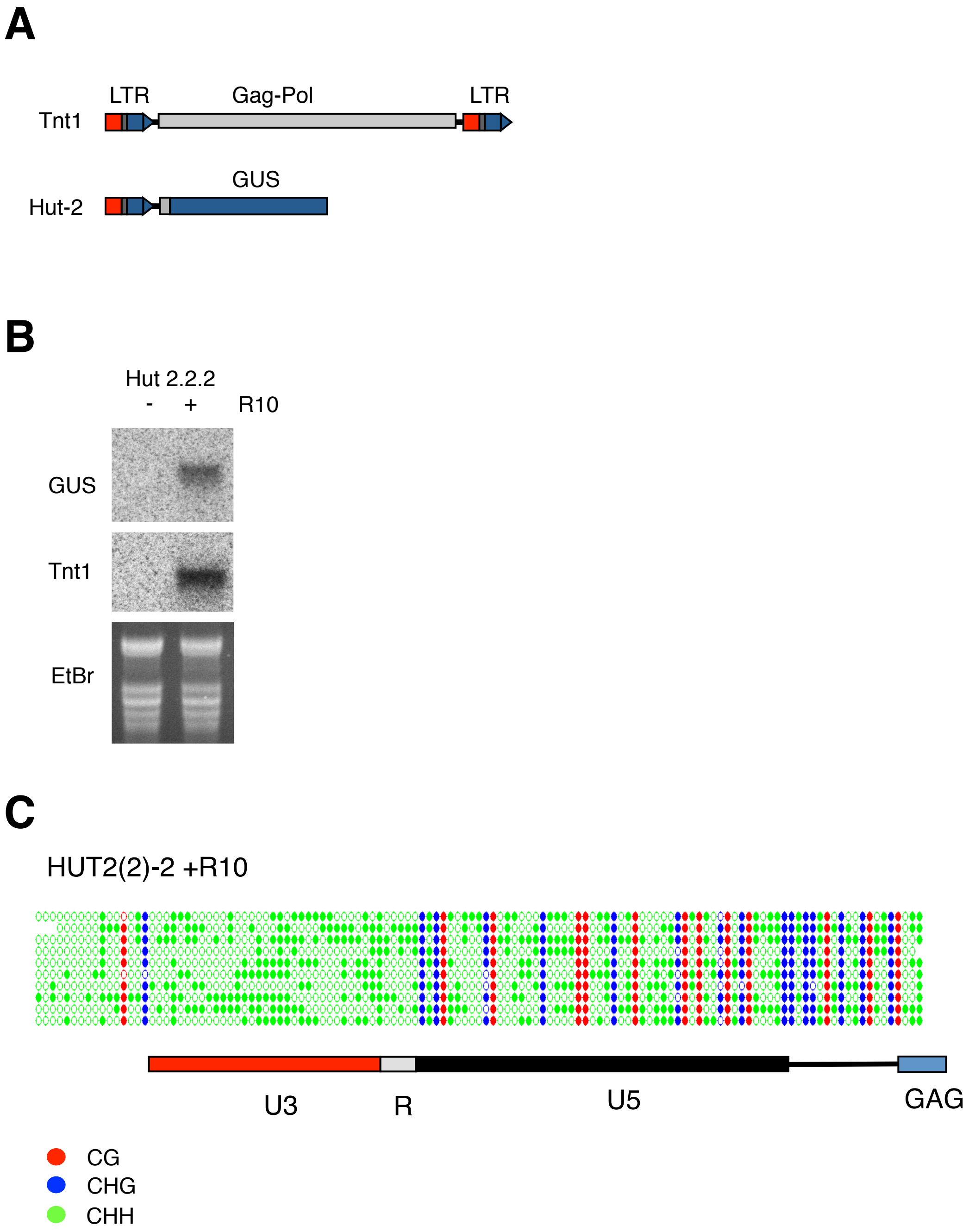

Supporting Figure S10. The Hut-2 transgene is not silenced when stably introduced in tobacco. (A) Schema of the Hut-2 transgene. A schema of the Tnt1 element is given for comparison. B) Northen blot analysis of the expression of a Hut-2 transgenic line in leaves non-treated (-) or treated (+) with R10. The hybridization with a Tnt1 probe and an image of the EtBr staining of the RNA gel are shown underneath as controls . The name of the line is given on top. C) Methylation analysis of the Hut-2 transgene. The 5' region of the Hut-2 transgene, including the 5' LTR, was amplified and sequenced from bisulfite converted DNA from R10-treated leaves. At least 10 clones were sequenced from each transgene (only one sequence is shown when the same sequence was obtained several times). The methylation state of each cytosine is shown as in Figures 3 and 4. The different regions of the transgene are shown under the sequence.
