# Supplementary material for: The Tnt1 Retrotransposon Escapes Silencing in Tobacco, Its Natural Host
Source: PLoS One. 2012 Mar 30;7(3):e33816. doi: 10.1371/journal.pone.0033816 (PMC3316501; doi:10.1371/journal.pone.0033816)
Supplement: Figure S11 — Constructs containing most of the internal sequence of Tnt1 can be expressed in tobacco transgenic plants. (A) Schema of the – END transgene. A schema of the Tnt1 element is given for comparison. B) Northern analysis of GFP expression in wild type plants agroinfiltrated with the –END construct and expressing it transiently (trans.), as well as in plants stably transformed with the same construct (the number of the transgenic line is indicated on the top). RNA was extracted from leaves treated (+) and non-treated (−) with R10, and probed with a GFP probe. An image of the EtBr staining of the RNA gel is shown underneath as loading control. The figure presents the analysis of 7 lines representative of the 36 obtained. (PDF) [file pone.0033816.s011.pdf]

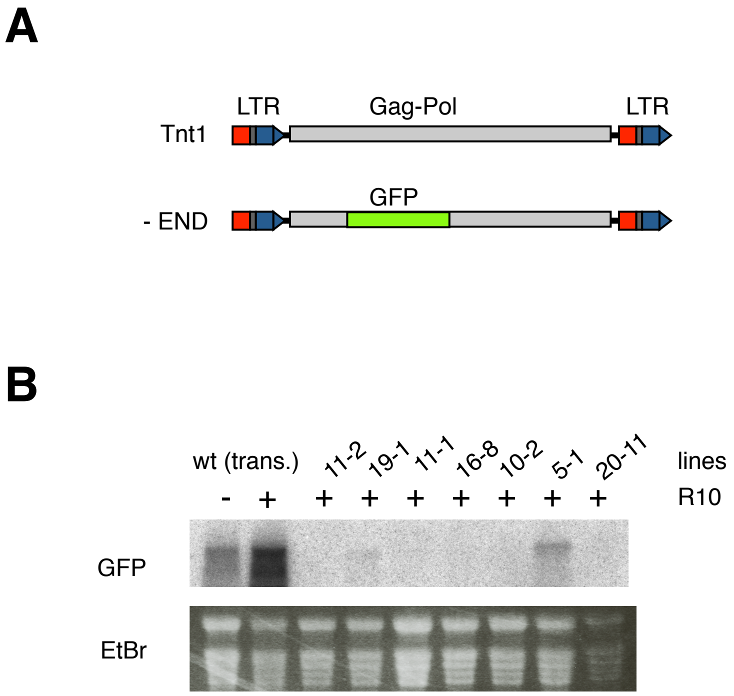

Supporting Figure S11. Constructs containing most of the internal sequence of Tnt1 can be expressed in tobacco transgenic plants. (A) Schema of the – END transgene. A schema of the Tnt1 element is given for comparison. B) Northern analysis of GFP expression in wild type plants agroinfiltrated with the –END construct and expressing it transiently (trans.), as well as in plants stably transformed with the same construct (the number of the transgenic line is indicated on the top). RNA was extracted from leaves treated with R10, and probed with a GFP probe. An image of the EtBr staining of the RNA gel is shown underneath as loading control. The figure presents the analysis of 7 lines representative of the 36 obtained.
