# Supplementary material for: The Tnt1 Retrotransposon Escapes Silencing in Tobacco, Its Natural Host
Source: PLoS One. 2012 Mar 30;7(3):e33816. doi: 10.1371/journal.pone.0033816 (PMC3316501; doi:10.1371/journal.pone.0033816)
Supplement: Table S1 — Primer pairs. (PDF) [file pone.0033816.s012.pdf]

Supplementary Table S1

Primer pairs for Bisulfite sequencing	
5'-GTGTTAAGTTTGTATGTTTGTAGGT-3'<br>5'-AACAACTCCAATAAAAAATTCTTCTC-3'	Transgene LTR-GFP-LTR
5'-GGTGGTAGTAATTTATAAGATTATGAGAG-3'<br>5'-AAACAAATCTCTCATCCTTCTTTACC-3'	Endogenous sequence FH391072
5'-TAATGAGAGTGTTTTTAAGGATGTTAGTG-3'<br>5'-AAACAAATCTCTCATCCTTCTTTACC-3'	Endogenous sequence s231f
5'-TGGTTTTTTGGAAATATTTTATTTTAG-3'<br>5'-AAACAAATCTCTCATCCTTCTTTACC-3'	Endogenous sequence s131b
5'-GGAGAGAAATATATATTGTATTTTTTGTAG-3'<br>5'-AAACAAATCTCTCATCCTTCTTTACC-3'	Endogenous sequence FH249215
5'-AAGAAGGGTATTTTTAAGTTTTTGG-3'<br>5'-AAACAAATCTCTCATCCTTCTTTACC-3'	Endogenous sequence FH271201
5'-TAATAATAGGATAAAATTTAAGTTTAGTT-3'<br>5'-TCCTTCTTTACCATATTAAAAAACC-3'	Endogenous sequence FH101327
**Primer pairs for ChIP**	
5'-CGGAAAGAGGTTATTCATTCAC-3'<br>5'-GGTGCTGAGAGAAGCTAAGATAG-3'	Actin
5'-CAGGCTCTTTAAAGGAACC-3'<br>5'-TTATACCTTGTCTGTGAAACC-3'	Transgene LTR-GFP-LTR
5'-ACATACGTGCTTATCAAGC-3'<br>5'-TTATACCTTGTCTGTGAAACC-3'	Endogenous sequence FH391072
5'-GACATCTTCCCACCATATC-3'<br>5'-TTATACCTTGTCTGTGAAACC-3'	Endogenous sequence s231f
5'-TGTTGAGTATTTGGTGAAGAG-3'<br>5'-TTATACCTTGTCTGTGAAACC-3'	Endogenous sequence s131b
**Primer pairs for Tnt1 hybridization**	
RT3: 5' - CCT AGA TCT TGA AGT G -3'<br>RT6: 5'- TCT CAT YTC ATC CAG AG - 3'	
